# Role of shock waves in materials processing: Fundamentals and Applications^☆^

**DOI:** 10.1016/j.ultsonch.2025.107473

**Published:** 2025-07-19

**Authors:** Abhinav Priyadarshi, Amanpreet Kaur, Mohammad Khavari, Justin A. Morton, Anastasia V. Tyurnina, Morteza Ghorbani, Paul Prentice, Jiawei Mi, Koulis Pericleous, Peter D. Lee, Dmitry G. Eskin, Iakovos Tzanakis

**Affiliations:** a School of Engineering Computing and Mathematics, Oxford Brookes University, Oxford, UK; b Department of Foundation Engineering and Physical Sciences, University of Nottingham, Nottingham, UK; c Mantisonix Ltd., Covent Garden, London, UK; d Brunel Centre for Advance Solidification Technology (BCAST), Brunel University of London, Uxbridge, UK; e Faculty of Engineering and Natural Science, Sabanci University, Tuzla, Istanbul, Turkey; f James Watt School of Engineering, University of Glasgow, Glasgow, UK; g School of Engineering, University of Hull, Hull, East Yorkshire, UK; h Department of Mathematics, University of Greenwich, London, UK; i Department of Mechanical Engineering, University College London, London, UK; j Research Complex at Harwell, Harwell Campus, Oxfordshire, UK

**Keywords:** Shock wave, Cavitation, Ultrasonic processing, Aluminium, Graphene, Composites

## Abstract

In recent years, ultrasonic processing (USP) technology has led to some of the most promising scientific breakthroughs in the field of pharmaceutical, food, environmental and material sciences leading to advancements in manufacturing, process efficiency, and material performance. However, the industrial scalability of USP still remains a key challenge, largely due to the lack of awareness, standardization and predictive multiphysics models. Optimizing this technology necessitates a bottom-up approach, emphasizing fundamental understanding of the physical phenomena at play prior to scaling-up. Despite the advancements of opto-acoustic characterization tools, the underlying root-cause driving these technological innovations remains unexplored. This paper provides a comprehensive overview of our work carried out in the last 5 years to uncover the fundamental mechanism that governs the deployment of USP in areas related to metal casting, additive manufacturing, production of nanomaterials and composites by employing *in-situ* high-speed visualizations techniques and characterization of acoustic emissions. The results presented and discussed in this article offer a new perspective on the pivotal role of cavitation-induced shock waves, shifting the focus from being just a by-product, to a primary driver of material modification during USP.

## 1 Introduction

Materials processing has undergone a paradigm shift over the past century, driven by the need for sustainable, energy-efficient techniques that enable precise structural control at macro, micro and nanoscales. This evolution has necessitated the fusion of multidisciplinary principles spanning fluid dynamics, thermodynamics, and materials science to engineer processes that balance scalability with minimal environmental footprint. Among such techniques, ultrasonic processing has emerged as a particularly promising approach, offering unique capabilities for material modification through sophisticated energy transfer mechanisms [[1], [2], [3], [4], [5], [6]]. This process operates across a broad frequency spectrum from 17 kHz to the MHz range, inducing dynamic multi-phase interactions within liquid media. At the heart of this technique lies cavitation, a stochastic phenomenon characterized by the formation, growth, and violent collapse of microscopic vapour/gas bubbles. Upon implosion, cavitation bubbles generate extreme conditions, including localised temperature spikes of several thousand Kelvins, high-impact liquid jets with ‘tip-hammer’ pressures in the MPa to GPa range and powerful shock waves (SWs) propagating at supersonic speeds [[7], [8], [9], [10], [11], [12], [13], [14], [15], [16]]. These intense effects can fundamentally alter material structures, damage nearby surfaces, or both. It is worth mentioning here that unlike cavitation driven processes, USP also encompasses several other solid-state methods, exploiting different physical mechanisms to achieve material transformation. For example, techniques such as ultrasonic shot peening [[17], [18], [19]], ultrasonic welding [20], ultrasonic machining [21], and ultrasonic additive manufacturing [22] rely on mechanical impacts, vibrations, and plastic deformation to modify material properties without phase transitions. This versatility allows researchers and engineers to select the most appropriate processing method based on the target material state and desired outcome, thereby expanding the scope of ultrasonic applications across diverse industrial sectors.

Cavitation, in general, induces a diverse range of physical and chemical dynamic phenomena in its surrounding medium or on an interface. Physically, they contribute to deformation [23], fragmentation [[24], [25], [26], [27], [28]], deagglomeration/dispersion [[29], [30], [31], [32]], degassing [1,5,[33], [34], [35], [36], [37], [38], [39], [40], [41]], erosion [8,[42], [43], [44], [45], [46], [47], [48], [49], [50], [51], [52]], wetting [53,54], crystallization [[55], [56], [57], [58], [59], [60]], exfoliation [[61], [62], [63], [64], [65], [66], [67], [68], [69], [70], [71]], emulsification [[72], [73], [74]], and atomization [[75], [76], [77], [78], [79], [80], [81], [82], [83], [84], [85], [86]]. Cavitation also accelerates chemical reaction kinetics through radical formation [[87], [88], [89]] and sonochemical activation [[90], [91], [92], [93], [94], [95]]. Depending upon the process mechanism(s) involved, these effects have been reported to be useful in applications ranging from surface cleaning [[96], [97], [98], [99]], wastewater treatment [95,[100], [101], [102], [103]], critical metals recovery and recycling [[104], [105], [106], [107]], lithotripsy, liposuction and cancer treatment [[108], [109], [110], [111], [112], [113], [114], [115], [116], [117], [118], [119]], food processing [72,86,[120], [121], [122]], drug delivery [[123], [124], [125], [126], [127], [128]], nanomaterial synthesis [68,70,71,92,94,129], microstructure refinement in metals and alloys [1,25,40,[130], [131], [132], [133], [134], [135], [136], [137], [138], [139], [140], [141], [142], [143], [144], [145], [146], [147], [148], [149], [150], [151]], fuel injection and sprays [[152], [153], [154]], to name a few. Studies have suggested that the mechanical effects from cavitation collapses in the form of SWs and liquid microjets play a crucial role in materials synthesis processes [[92], [93], [94],^129^,^155^,^156^]. As the SWs dissipate energy at their advancing fronts, they can induce localized heating and phase transitions in the surrounding liquid medium. This energy dissipation can also modify immersed substances, potentially leading to chemical or structural changes in the materials. In spite of extensive research and development in the aforementioned applications, fundamental mechanisms governing the response dynamics phenomena are not very well understood and have only been hypothesized and theoretically studied over the years leaving a crucial gap in the literature.

Moreover, in order to maximize the efficacy of USP, process optimization becomes crucial, which can require balancing of the contributions from non-inertial and inertial cavitation regimes through careful tuning of ultrasonic parameters (e.g. sonotrode size, frequency, amplitude/input power and sonication time) and liquid properties (e.g. viscosity, vapour pressure and surface tension) [79,102,157]. Equally important is the fine tuning of process design elements, such as vessel geometry and sonotrode positioning that govern acoustic pressure uniformity, for consistent material treatment that boosts efficiency and product quality [158,159]. Temperature control is another crucial aspect that plays a pivotal role by balancing cavitation intensity with energy dissipation, directly impacting material production and properties [160]. These parameters allow precise manipulation and generation of high energy SWs within the medium improving process efficiency (e.g., faster production rates, reduced energy use) and material performance (e.g., refined grains, spherical powders, defect-free composites). While foundational studies have characterized single bubble dynamics in idealized systems [7,8,[161], [162], [163], [164], [165], [166]], real-world material processing often involves bubble clusters, SWs interference and absorption, and the nonlinear interactions with immersed solids or liquids [[167], [168], [169], [170]]. Recent advancements in real-time diagnostic tools such as ultra high-speed imaging, synchrotron X-ray radiography, and acoustic characterization have started to unravel these complexities. Nevertheless, a systematic framework for tailoring the beneficial effects of SWs to specific applications remains elusive, hindering their industrial scalability.

**Fig. 1:**
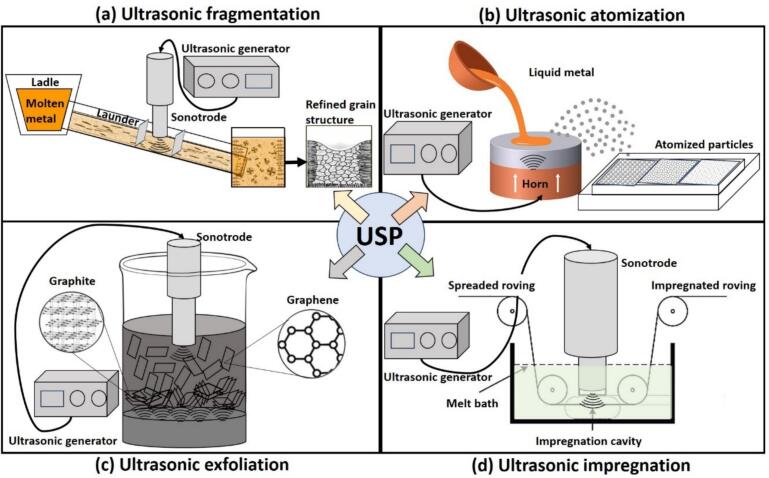
Schematic illustrations of ultrasonic processing applications for (a) grain refinement via fragmentation, (b) metal powder production via atomization, (c) graphene synthesis via exfoliation, and (d) fiber dispersion in composites via impregnation.

This article reviews the work carried out by our research groups in the last five years to elucidate the role of cavitation-induced effects in materials processing. By integrating state-of-the-art experimental tools, we have established mechanistic links between SW dynamics and material response across four key applications: (1) ultrasonic grain refinement in metallic alloys; (2) ultrasonic atomization of metal powders; (3) ultrasonic liquid-phase exfoliation (ULPE) of graphite into graphene; and (4) fiber impregnation in polymer composites. We have dissected these applications (schematically shown in Fig. 1) through state-of-the-art *in-situ* opto-acoustic tools involving high-speed imaging and acoustic pressure detection, which collectively resolved SWs interactions at macro and micro scale resolutions using a transparent liquid analogue (water). In this review, we will first delve into uncovering the fundamentals of cavitation-induced SWs generation using *in-situ* characterization in different liquids in a range of input powers and temperatures. Subsequent sections correlate these fundamentals to new underlying physics, highlighting process-specific mechanisms and linking to efficacy. Finally, we close this review by summarizing the most relevant findings that address the critical role of cavitation-induced SWs for optimizing material processing using ultrasound, and outlining challenges, promising future research directions and methods that can be optimized based on a fundamental understanding of process mechanisms. Through this work, we also aim to bridge mechanistic understanding with industrial scalability and advancements, paving the way for a new era of material modifications through USP.

## 2 Spatio-temporal characterization of shock waves

Cavitation-induced SWs underpin numerous industrial, medical, and scientific applications by generating intense, localized pressure fields that can be finely manipulated to drive USP. The fundamental characteristics of these SWs stem from the rapid collapse of bubble clusters, and depend critically on the properties of the liquid medium including viscosity, surface tension and density, together with the conditions under which they are generated [171,172]. In this section, we will discuss a series of experimental observations that reveal their spatial and temporal characteristics through *in-situ* high-speed imaging and acoustic pressure measurements, while also examining the influence of different liquids, temperature, ultrasonic input power on the dynamics within and around the cavitation zone.

**Fig. 2:**
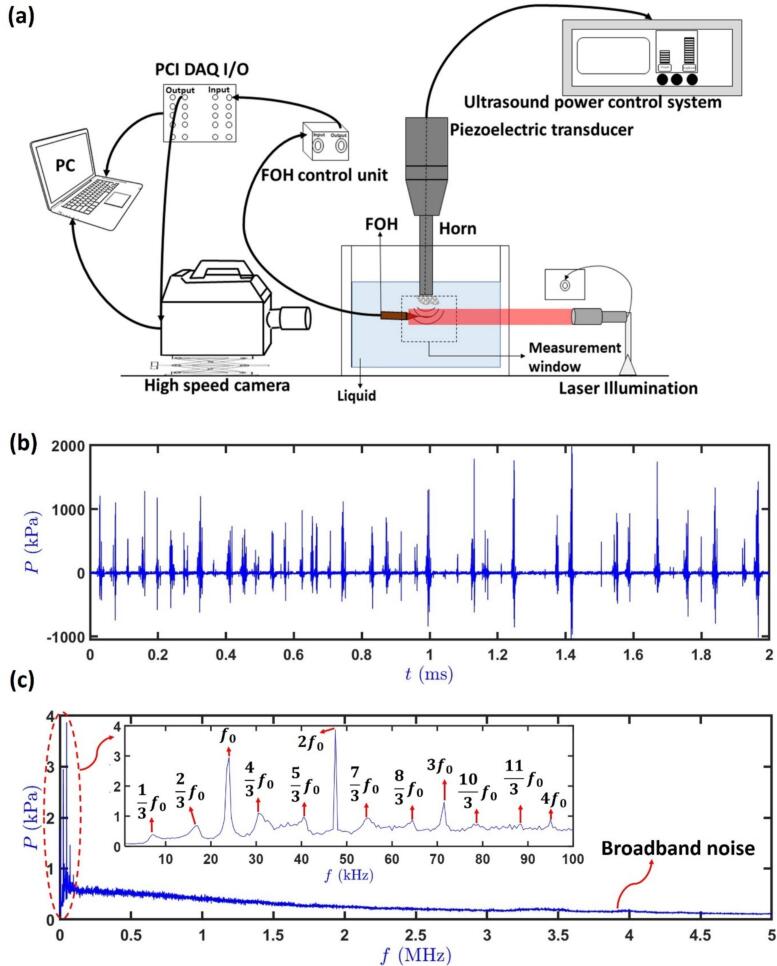
(a) Schematic of the experimental setup used for qualitative (high-speed visualization) and quantitative (acoustic measurement) characterization of shock waves, (b) pressure–time profile of cavitation-induced SWs in DIW at 60% ultrasound input power with a 3-mm horn tip, showing distinct major and minor peaks, and (c) corresponding acoustic spectrum, displaying fundamental frequency, harmonics, sub and ultraharmonics, and broadband noise up to the MHz range.

The cavitation intensity can be adequately elucidated in terms of the induced acoustic pressure [157,[173], [174], [175], [176], [177]], and, therefore serves as a perfect quantitative tool in characterizing the SWs dynamics. Absolute measurement of acoustic pressure requires the use of calibrated hydrophones. The resulting raw voltage–time data from the hydrophone is deconvoluted to obtain the actual pressure waveforms and to determine the magnitudes of pressure fluctuations. Measurements are then quantified using the maximum pressure (*P*_max_) and root-mean-square pressure (*P*_RMS_) values, averaged over multiple waveforms to get a reliable measure. Fibre optic hydrophones (FOH) are often employed because of their broad frequency range, allowing them to capture prominent spectral features generated by SWs. Details of the FOH used for characterizing SWs can be found elsewhere [178,179]. Apart from hydrophones, SWs can also be qualitatively described through ultra-fast high-speed camera, configured with collimated pulsed illumination. The experiments described in this review involved an FOH developed by Precision Acoustics Ltd. attached to a holder inside a glass tank. With a calibrated range up to 30 MHz, the FOH provided broad omnidirectional response. Sonication was applied by a 24 kHz transducer (UP200S, Hielscher Ultrasonics GmbH) attached to a cylindrical Ti sonotrode (*Φ* 3 mm) in deionized water (DIW) and various organic liquids at three power levels (20 %, 60 %, and 100 %) across various positions within the vessel at room temperature. The corresponding peak-to-peak amplitude of the sonotrode tip ranged from 42 – 210 µm. The FOH was placed within the measurement window of −10 ≤ *x* ≤ 10 mm and 1 ≤ *y* ≤ 10 mm, with the centre of the sonotrode tip taken as the origin (0, 0). The acoustic emissions were recorded using a digital oscilloscope (PicoScope-3204D, Pico Technology). Prior to each ultrasound run, baseline background acoustic signals (typically comprising electronic, environmental, and fluid dynamic noise) were recorded with the hydrophone in the same position and medium. These signals were then subtracted from the active ultrasound recordings in time-domain to isolate cavitation-induced acoustic emissions. This denoising technique improves signal clarity and is effective for identifying key spectral features such as harmonics, subharmonics, ultraharmonics and broadband noise after deconvolution of the resultant signal, as clearly shown in Fig. 2c. The analysis of experimental data employed an in-house MATLAB code for deconvolution of the raw data, as described in our previous works [177,178,180]. This code computes the required pressure magnitudes in the time domain from the original voltage data, via deconvolution of the hydrophone response with the broadband calibration data. Ultra-fast imaging using HPV X2 high-speed camera (Shimadzu, Japan) was conducted at frame rates up to one million per second, with images captured at 400 × 250 pixels and an exposure duration of 200 ns. Illumination was provided by a CAVILUX Smart UHS system (Cavitar Ltd), which emitted 10 ns laser pulses at 640 nm, enabling visualization of the SWs generated by collapsing bubbles (Fig. 2a).

Fig. 2b shows a typical example of a pressure–time profile obtained at 60 % ultrasound input power exhibiting distinct major and minor peaks, where major peaks correspond to periodic collapses of large bubble clusters generating high-pressure SWs, while minor peaks arise from emissions at the driving frequency superimposed by vigorous sub-cluster and satellite bubbles oscillations. The horn tip was submerged 10 mm below the liquid surface and located in the centre of the glass tank, while FOH was positioned at x  = -1 and y = 3 mm within the measurement window relative to the sonotrode centreline axis. Peak pressures (*P*_max_) reached up to 2 MPa at a distance of 3 mm from the origin, with an average *P*_RMS_ of 0.4–0.5 MPa, indicating strong cavitation activity. The acoustic noise spectrum in Fig. 2c revealed a broad range of components, including fundamental frequency (*f*_0_) and its harmonics (2*f*_0_, 3*f*_0_, etc.), alongside subharmonics (e.g., *f*_0_/3, 2*f*_0_/3) and ultraharmonics (e.g., 4*f*_0_/3, 5*f*_0_/3, etc.). The presence of ultraharmonics, subharmonics and broadband noise in the MHz range indicate non-linear bubble interactions and the presence of SWs originating from violent bubble cluster oscillations and subsequent collapses [[181], [182], [183], [184]]. The gradual decay in spectral amplitude at higher frequencies can be linked to energy attenuation across higher frequencies, with most energy concentrated in the low to mid-frequency range (up to 100 kHz). These spectral characteristics indicated a combination of periodic and inertial effects (chaotic cavitation collapses) of varying pressure amplitudes, making the resulting SWs highly relevant for materials processing applications.

**Fig. 3:**
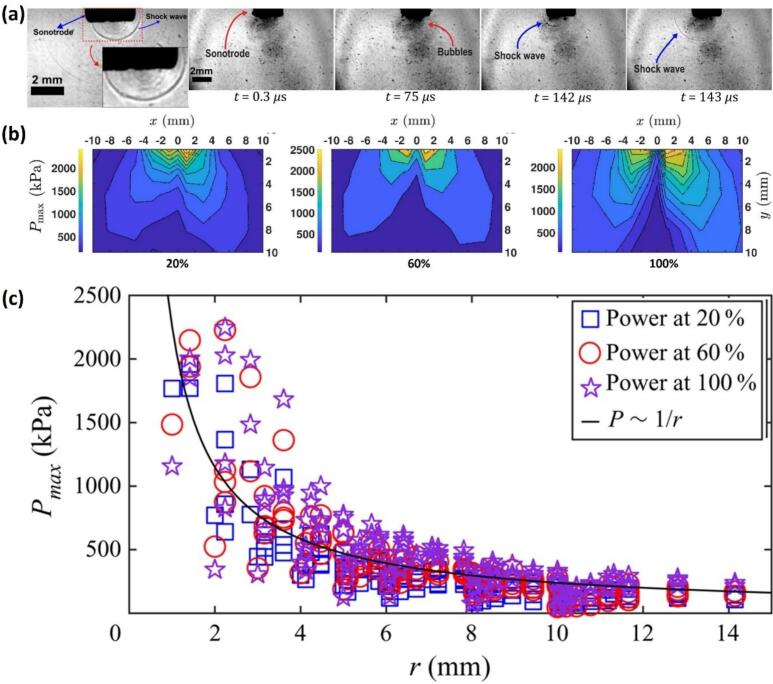
(a) High-speed image sequence showing SWs emission and propagation into the DIW bulk liquid from the sonotrode at different time intervals. (b) Contour maps of shock pressure (P_max_) across the x-y plane for different transducer power levels, showing a strong pressure gradient with peak values near the sonotrode. (c) Shock pressure as a function of radial distance (r), demonstrating a 1/r decay trend. After [178].

In Fig. 3a, snapshots depict the emission of SWs at different time intervals obtained from high-speed imaging in DIW. Notably, SWs emitted near the sonotrode edge propagated unhindered to the bulk liquid, influencing pressure distribution. Fig. 3b shows the contour mapping of the recorded maximum pressure *P*_max_ across all horizontal (*x*) and vertical positions (*y*) for three different transducer powers. The plots show that *P*_max_ was highest near the sonotrode, decreasing significantly with distance, which is attributed to energy dissipation during shock front propagation. Within a 10 mm range, *P*_max_ dropped by 75–78 % for all input powers, emphasizing the proximity-dependent nature of pressure magnitudes. The acoustic pressure data fit well with 1/*r* scale (Fig. 3c), where *r* is radial position measured from the source (*r* = √(*x*^2^ + *y*^2^)), which is consistent with previous predictions [9,10,185,186]. For example, at 100 % ultrasound power in water, *P*_max_ drops from ∼ 2 MPa near the sonotrode to below 0.5 MPa at 10 mm, underlining the localized nature of SWs propagation.

**Fig. 4:**
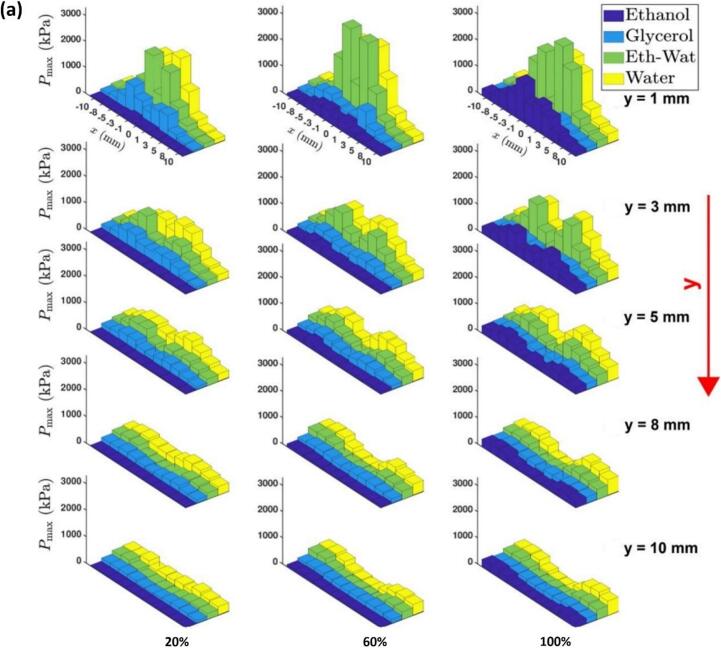

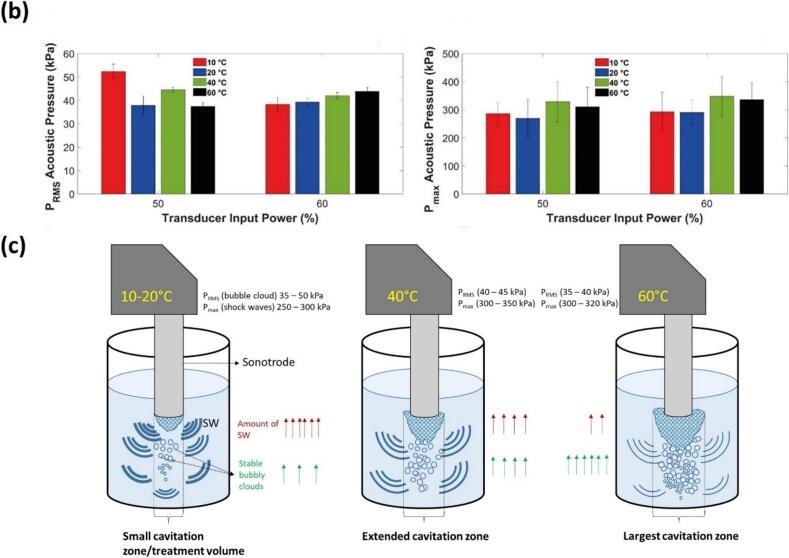
(a) Three-dimensional bar plots showing the spatial distribution of shock pressure (P_max_) for different working fluids in the cavitation zone (y indicates the distance from the origin, i.e. centre of the horn tip), (b) bar graphs depicting P_RMS_ and P_max_ for water at different temperatures, for 50 % and 60 % transducer input powers with a 22 mm horn tip, measured using FOH at 2.5 cm away from the origin, and (c) schematic representation of cavitation activity and their corresponding shock pressure conditions signifying the relationship between temperature and the extent of cavitation zone. After [65,179].

This decay varies with ultrasound power and liquid properties; in less viscous media like ethanol, *P*_max_ is reduced by up to 90 % compared to water, reflecting increased energy absorption and bubble damping (see Fig. 4a). The shock pressure amplitude is largely influenced by the speed of a SWs, which is highest near the source bubble (∼4000 m/s), reaching approximately 2500 m/s within just 100 μm of the source, before decreasing to the speed of sound as it propagates further into the medium [13,165]. These pressure bar plots (Fig. 4a) and contour maps (Fig. 3b) also indicated non-symmetrical cavitation zones with acoustic pressure field being strongest near the source, and its spatial distribution being affected by factors such as bubble cloud shielding and the geometry of the cavitation zone [175,177,187]. These results indicate that measurement in the immediate vicinity of the acoustic source is crucial for understanding the dynamics of the generated SWs.

Judicious choice of liquid medium can further enhance these SWs characteristics. Fig. 4a shows the spatial pressure distribution of shock pressure (*P*_max_) for four liquids with largely different physical properties across multiple vertical and horizontal positions within the vessel measured at varying power levels of the ultrasound. For example, in highly viscous liquids such as glycerol, the formation of collapse shocks is often suppressed, and cavitation can occur through shockless rebounds [188]. High viscosity also causes rapid attenuation of SWs, restricting the development of a full cavitation zone [157]. In contrast, low-viscosity liquids are dominated by surface tension (γ) and inertial forces, which promote cavitation activity. For example, ethanol (with γ approx. 0.022 N/m at 20 °C) tend to have longer-lived bubbles that resists strong bubble collapse, reducing SWs generation. Water with higher γ (approx. 0.079 N/m at 20 °C) supports more intense bubble collapses with stronger SWs generation thus showing the strongest peak pressure below the horn tip, whereas an ethanol–water mixture and ethanol showing almost 90 % decay in the shock pressure amplitude. Similar relationship exists for vapour pressure and density. Liquids with higher vapour pressure lead to the formation of numerous, long-lived bubbles which cushion the propagating SWs. A high density of cavitation bubbles near the source reduces the transfer of ultrasonic energy into the bulk liquid, leading to weaker SWs. For example, in an ethanol–water solution, a mist-like pattern of tiny bubbles was observed, which absorbed the SWs propagation, and resulted in stronger shielding. In this case, the shielding factor decreased by 43.5 % at 100 % power compared with the value at 60 % power, suggesting increased collapsing events of the tiny bubbles in the mist pattern. The shielding effect was shown to increase with input power in most liquids, but decrease in viscous glycerol.

Beyond the inherent differences in physicochemical properties across liquids, the temperature of a liquid also significantly influences SW characteristics through changes in the bulk properties (e.g., viscosity, surface tension, vapour pressure, and gas solubility), which in turn affects its cavitation intensity. Understanding this relationship is crucial for optimizing processes relying on acoustic cavitation [42]. For this experiment, the FOH was deliberately positioned at a distance of 2.5 cm from the acoustic source to ensure that the recorded acoustic pressure signals were representative of the bulk liquid behaviour and not influenced by the localized heating and non-linear and cavitation shielding effects near the sonotrode tip. Fig. 4b shows that water temperature close to 40 °C exhibited the most aggressive cavitation activity despite registering lower *P*_RMS_ in comparison to 10 °C. This is mainly because the cavitation bubble dynamics involving shock pressure spikes, size of the cavitation zone and amount of SW fronts changes with respect to changes in temperature, as schematically illustrated in Fig. 4c. While lower temperature induced large pressure magnitudes, the bubble cloud collapses were more confined within a localised region supressing the number of SW fronts released near to the source [65]. Whereas at temperatures above 50 °C, the spatial distribution of bubbles increased significantly leading to substantial acoustic shielding. Thus, a trade-off temperature range, where the cavitation activity is both aggressive and evenly distributed was found, at around 40 °C. At elevated temperatures, vapour pressure of the liquid also increases with temperature, leading to increased cloud formation, which has important implications for the design of USP systems/reactor. It can also be observed from Fig. 4b that *P*_RMS_ did not significantly differ between all temperature levels, particularly at 40 °C for both 50 % and 60 % input powers, where they were almost identical. This comparability implies a certain degree of flexibility in choosing the appropriate power setting at optimal temperatures, as similar acoustic pressures can be achieved. Thus, the ideal parameters for efficient USP include using water at 40 °C, with an input power between 50–60 % (peak-to-peak amplitude ranging from 23 – 27 µm for 22 mm horn tip), and a driving frequency in the range of 20–24 kHz. Such a setup ensures an optimal balance between cavitation intensity and SW propagation while minimizing shielding and excessive bubble formation that diminishes SW power.

Therefore, strong SWs and effective cavitation conditions for material synthesis and processing requires careful optimization of ultrasound power, liquid properties and treatment temperature to precisely control SWs intensity and maximize their effectiveness as summarized in Table 1. In the following sections, we explore the role of SWs in processing a range of materials, beginning with grain refinement in aluminium alloys, followed by aluminium powder atomization, graphene production, and concluding with the development of composite materials.

**Table 1:** Influence of sonication parameters and liquid properties on cavitation and SWs characteristics. After [189].

**Parameter**	**Condition**	**Shock wave intensity**	**Cavitation zone**	**Bubble dynamics**	**Acoustic shielding**	**Cavitation characteristics**
**Input Power**	Low	Low (limited collapse of bubbles)	Small and localized	Few transient bubbles with limited energy release	Minimal (lower bubble density reduces interference)	Gentle cavitation suitable for mild dispersion or delicate materials
	High	High (violent bubble collapse generates strong SWs)	Large and widely distributed	High density of transient bubbles, frequent collapses produce strong mechanical effects	Significant (overlapping bubbles attenuate ultrasound and SWs)	Aggressive cavitation suitable for rapid treatment
**Temperature**	Low	High (due to formation of vapour bubbles that collapse easily)	Restricted under the tip of the sonotrode	Vapour and transient bubbles form easily; however, a high surface tension resists collapse, especially when the surface tension/viscosity ratio is high	Minimal (fewer non-inertial bubbles present)	Promotes strong cavitation, enhances SW generation in confined zone
	High	Low (non-inertial gas bubbles resist collapse)	Extended cavitation zone with numerous bubbly clouds	Predominantly gas bubbles form due to decreased solubility, less resistant to collapse due to low surface tension	Moderate (cushioning effect leading to absorption of SWs)	Weak cavitation, reduces mechanical effects but promote acoustic streaming instead
**Viscosity**	Low	High (intense collapses)	Broad (strong propagation into the bulk liquid	Transient bubbles collapse violently	Moderate (dense bubble field absorbs some energy)	Strongest cavitation below horn tip
	High	Low collapse (shock are suppressed)	Severely restricted, rapid attenuation of SWs	Cavitation occurs via shockless rebounds (collapse is minimal)	Low (fewer collapses reduces shielding)	Weak cavitation activity, shielding decreases with power
**Surface tension**	Low	Low (bubbles less prone to collapse)	Extended cavitation zone	Long lived bubbles absorb SWs energy	High (attenuation and absorption of SWs)	Cavitation is cushioned, significant decay in SWs intensity
**Vapour pressure**	High	Low (numerous gas bubbles cushion SWs)	Extended cavitation zone	Stable bubbles persist	High (absorption of SWs)	Increased bubble population leads to stronger shielding

## 3 Metal casting: Cavitation induced fragmentation and grain refinement in Al alloys

Grain refinement in Al alloys is a critical process that significantly enhances the mechanical properties of the final cast product, such as strength, ductility, and resistance to hot cracking. Many established grain refinement strategies rely on promoting heterogeneous nucleation by adding suitable grain refiners to the melt, which is controlled by their high wettability, matching crystal structure, and the dimensions of the inoculant particles [190]. However, achieving uniform and evenly distributed activated nucleation sites throughout the melt can be challenging, and thus, may not fully account for the dynamic solidification process.

USP presents a dynamic and active method to refine grain structure by directly targeting the fragmentation of evolving dendritic and intermetallic phases within the solidifying alloy [24,45,133,138,141,145,146,[191], [192], [193], [194], [195], [196], [197], [198], [199], [200]]. Under normal casting conditions, primary intermetallics such as Al_3_Zr crystals in Al-Zr alloys can grow to considerable sizes and elongated shapes. These large crystals are often brittle and act as stress concentrators, leading to cracks and premature failure, ultimately reducing the alloys’ ductility and mechanical performance. USP serves as a promising technique to refine these intermetallics by inducing cavitation, which generates SWs and high-speed liquid microjets thereby fragmenting the crystals into smaller particles [25,132,140,141,201]. These fragmented particles (typically in the range of 1 – 5 µm) then act as potent nucleation sites for aluminium, leading to the formation of a fine, equiaxed grain structure.

In this section, we discuss a series of experiments to delineate the crystal fragmentation process during ultrasonic treatment of Al alloys using water as a transparent analogue as it possesses closest cavitation properties similar to liquid Al [4,157,176]. The Al_3_Zr crystals used in these experiments were chemically extracted from the solidified ingot of an Al-3 wt% Zr alloy under slow cooling conditions without USP as explained in [193]. This was deliberately done in order to extract large crystals with sizes in the range of 2–5 mm. The extracted crystals were found to exhibit layered and faceted morphology with pre-existing cracks (of the order of tens to hundreds of microns) caused by the residual stresses arising during its solidification and extraction [194]. To understand their fragmentation mechanism, experiments were conducted using both single cavitation bubble and cloud of bubbles. The former involved generating a controlled cavitation bubble using a focused laser pulse, while the latter utilized a high-frequency (24 kHz) ultrasonic transducer attached to a 3-mm Ti sonotrode to generate a cloud of bubbles (similar to the setup described in the previous Section 2). High-speed imaging was employed to capture the interaction between the cavitation bubbles and the intermetallic crystals in real-time, for both configurations [24,199]. Acoustic pressure measurements were taken using a calibrated FOH to characterize the SWs intensity generated during bubble collapse, enabling the analysis of impact pressures and developed stresses required to break these crystals. We also characterized the cavitation activity within liquid aluminium using state-of-the-art high-temperature cavitometer pressure sensors calibrated across a broad frequency spectrum of 8–400 kHz in the National Physical Laboratory (UK), within vessels with resonant (*L*, 2*L*) and non-resonant dimensions (0.5*L,* 0.7*L*), where resonance length *L* is equal to wavelength *λ* of the ultrasonic wave, in the tested medium [157,180,202]. This was done to understand the effect of vessel geometry on cavitation intensity and grain refinement efficiency. Following this, experiments were conducted to evaluate the impact of USP during Direct-Chill (DC) casting of an AA6XXX alloy in a pilot scale facility. The efficiency of the process was then gauged through structural observations at both micro and macroscale, both in the presence and absence of ultrasound.

**Fig. 5:**
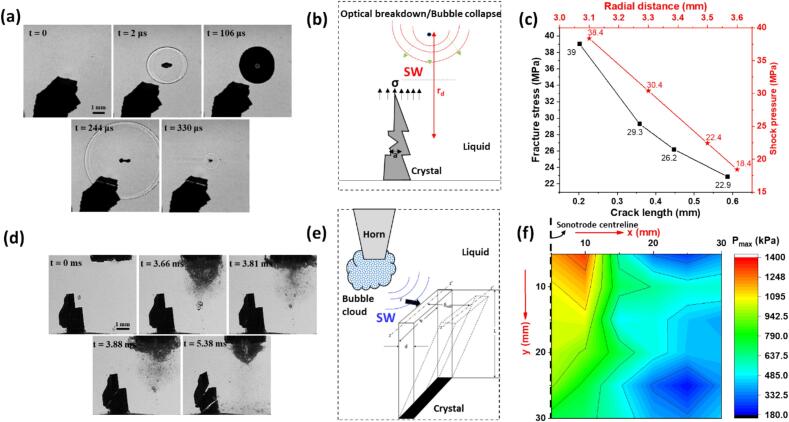
(a) Sequential images captured at 500 kfps showing in-situ Al_3_Zr crystal fragmentation caused by LIB with (b) bubble breakdown and collapse occurring at a radial distance r_d_ from the notched crystal, (c) corresponding shock pressure decay with radial distance and critical stress needed for crystal fragmentation at different crack lengths, (d) high-speed image sequence captured at 100 kfps illustrating crystal fragmentation by a cloud of collapsing bubbles (e) in a cantilever configuration, and (f) FOH measured shock pressure responsible for low cycle fatigue loading and fracture (black dashed line indicates sonotrode’s centreline). After [193,194].

From Fig. 5a, it can be seen that crystal fragmentation was a cumulative response to SWs from the laser bubble breakdown and collapse phases. No direct physical interaction with the crystals occurred apart from the interaction of SWs with the intermetallic, and fragmentation took less than a millisecond. The asymmetrical collapse of the bubble replicated real shock pressure conditions, leading to the instantaneous brittle fracture of the crystal. Vogel et al. [165] theoretically approximated the variation of pressure amplitude (*P*_r_) of the propagating shock front emitted from spherical bubble collapse with respect to the radial distance (*r*_d_) measured from the optical breakdown to the crystal location (Fig. 5b), which is given by:

(1) $$P_{r}=c_{1}\rho u\left(10^{\left(u-c_{s}\right)/c_{2}}-1\right)+P_{h}$$

where *P*_h_ is the hydrostatic pressure, *ρ* is the density of the liquid medium, i.e., water prior to SWs emission, *u* is the SWs velocity estimated from the time derivative function *r*(*t*), *c*_s_ is speed of sound in the medium, and *c*_1_ and *c*_2_ are constants derived empirically as 5190 m/s and 25306 m/s, respectively. Shock pressure estimated using Eq. (1) ranged from 20 to 40 MPa for different radial distances of the crack tip to the bubble centre. The critical stress (*σ*) inflicted by the SWs for the intermetallic fragmentation was further calculated based on the Griffith criterion for a crystal with pre-existing crack of different initial lengths assuming the mode I fracture, given as:

(2) $$K_{\mathrm{IC}}=C\sigma \sqrt{\pi a}$$

where *K*_IC_ is the fracture toughness of the crystal measured to be approx. 1.1 MPa√m [199], *C* is the crack size dependent constant. Fig. 5c shows that the shock pressure released from laser induced bubble (LIB) collapses were larger to that of critical stress necessary to induce brittle fracture of the intermetallic crystal. However, during continuous cavitation experiments, the fragmentation process was more complex, involving multiple bubbles and their interactions. The Al_3_Zr crystal (positioned away from the cavitation zone) was subjected to a cloud of cavitation bubbles generated by a 24 kHz ultrasonic transducer. High-speed imaging at 100,000 frames per second (fps) showed that the crystals initially underwent low-cycle fatigue loading due to the cyclic pressure exerted by the stresses from SWs and acoustic streaming from the pulsating bubbles (Fig. 5d). This was followed by catastrophic brittle failure as the cumulative stress from the SWs exceeded the fracture toughness of the crystal. The maximum shear stress (*τ*_max_) induced at the tip of a similar crystal was determined by considering a simple cantilever beam model (Fig. 5e) with maximum free-end deflections (*δ*_max_) caused by propagating SWs at different exposure time (acoustic cycles) is shown in Table 2, as described by the following equation:

(3) $$\tau_{\max}=\frac{9\delta_{\max}EI}{2L^{3}db}$$

where *E* is the elastic modulus of the crystal (∼200 GPa), *I* is the moment of inertia, *L*, *d* and *b* are length, thickness and width of the crystal, respectively. The shock pressure generated by the collapsing bubble clouds was measured by FOH and found to be up to approx. 1.5 MPa, which, although lower than in the single-bubble case, was sufficient to induce fatigue failure over multiple acoustic cycles (Fig. 5f). Notably, continuous ultrasonic cavitation experiments generated shock pressure fields approximately ten times lower than those predicted by Vogel’s approximation. This difference likely stemmed from the acoustic shielding effect, where shock waves from collapsing bubble clouds are absorbed and dampened by surrounding bubbles in the cavitation zone. Additionally, the higher shock pressures in LIB collapses emerged from the significantly larger bubble size. So, fragmentation observed in continuous cavitation resulted from the cumulative and repetitive impact of shock waves on the crystal, in contrast to the more intense implosion of a single bubble. In real melts, however, the crystals are often free-floating and are therefore broken down by both SWs and acoustic streaming effects. Acoustic streaming helps to redistribute the fragmented crystals and ensures they are exposed to the cavitation zone for further refinement [4,141]. We found out that the crystal fragmentation process initially occurs rapidly and then slows down, during which extended sonication reduces fragment size and increases the relative density of the fragments [194].

**Table 2:** Maximum shear stress (*τ*_max_) produced at crystal’s free end estimated using Eq. (3) for deflection (*δ*_max_) measured at different time intervals [203].

**Time (ms)/Acoustic cycles**	***δ*_max_ (µm)**	***τ*_max_ (kPa)**
1.05/25	24.3 ± 1.6	141 ± 15
3.62/87	48.7 ± 1.4	280 ± 58
5.07/122	73.1 ± 1.2	423 ± 87
5.28/127	97.5 ± 1.5	565 ± 114

**Fig. 6:**
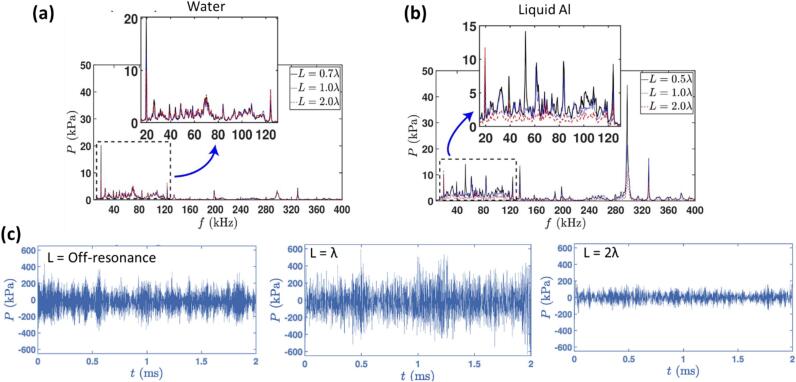
Measured acoustic pressure in frequency domain for (a) water and (b) liquid Al and, (c) time domain for liquid Al obtained under resonant and non-resonant vessel conditions using a bespoke high-temperature cavitometer. After [180].

One of the key challenges of USP, particularly in the processing of liquid aluminium is scaling up [5,176]. Thus, optimising process parameters such as acoustic intensity, melt temperature, cavitation zone, acoustic streaming and launder design with strategically positioned baffles downstream to control the residence time of the melt within the cavitation zone becomes crucial to maximize the treatment of the alloy. To address this, we conducted initial experiments in the bulk melt to determine the effectiveness of this process by identifying the optimum process parameters [[204], [205], [206]] and the optimal vessel dimensions that intensifies cavitation activity leading to resonance within the tank resulting in higher pressure amplitudes [180]. It was found that the resonance length of the processing vessel significantly impacts the acoustic pressure magnitude in liquid aluminium. The pressure magnitude in liquid Al was sensitive to resonance length, whereas in water it was not (refer to Fig. 6a and b). It is worth noting that the differing cavitation behaviour between water and liquid Al may also be attributed to the substantial difference in their vapour pressures at operational temperatures. Water, with a relatively high vapour pressure at room temperature, readily supports cavitation bubble growth. In contrast, molten aluminium at ∼ 700 °C exhibits an extremely low vapour pressure, implying that cavitation in aluminium is more dependent on the diffusion and presence of dissolved hydrogen. This fundamental difference may contribute to the observed sensitivity of cavitation intensity to resonance length. The response in liquid aluminium also exhibited significant pressure signatures under the sonotrode at subharmonic and ultraharmonic frequencies (associated with SWs emissions) and high-frequency peaks (around 300 kHz) indicating the presence of sustained pulsating bubbles as shown in Fig. 6b and experimentally observed through synchrotron observations [207,208]. Thus, a broader range of frequencies become important for characterizing different cavitation regimes and quantifying acoustic pressures. Additionally, it helps in better understanding how cavitation bubbles behave differently in liquid Al and water affecting cavitation zone [204,206], acoustic streaming [205,209] and pressure fields [177]. For example, the Minnaert equation [[210], [211], [212]] predicts resonant bubble radii in liquid Al to be 10 – 50 μm. However, synchrotron observations [207] showed sizes in a narrower range of 15 – 20 µm aligning well with the observed peaks at 300 kHz. This agreement further emphasizes that the cavitation activity in liquid Al is not only influenced by the shock pressure conditions from bubble implosions but also shaped by resonant modes and spatial pressure distributions from non-inertial pulsating bubbles, with important implications for industrial cavitation treatment processes.

Fig. 6c shows the pressure distribution in the time domain for liquid aluminium in different tank lengths. It can be clearly observed that the pressure fluctuations and cavitation activity varied significantly depending on the vessel dimension. For the off-resonance length, the pressure fluctuations were visible but with lower magnitude compared to the resonance length of *L = λ*, typically ranging up to 400 kPa. The waveform in the off-resonance tank showed less pronounced peaks and troughs, indicating less efficient cavitation suggesting that the acoustic waves do not constructively interfere, leading to weaker cavitation activity. At *L = λ*, pressure fluctuations showed greater intensity, indicating enhanced cavitation activity with increased amplitudes of up to 600 kPa. The pressure oscillations in the double-resonant vessel (2*λ*) were also regular but less pronounced than in the resonant vessel (*λ*), with amplitudes ranging up to 200 kPa. This indicates that a larger tank does not lead to enhanced cavitation even under resonance conditions, which may be related to reduced bubble volume fraction and standing wave formation thereby reducing overall cavitation intensity.

**Fig. 7:**
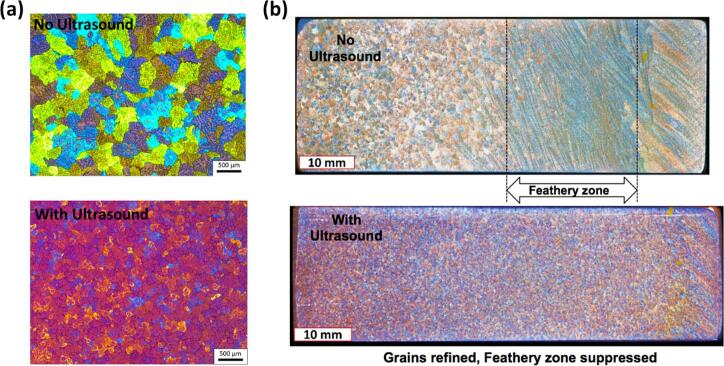
(a) Anodized microstructures of pure Al and (b) macrostructures from a DC casting of 6XXX-series alloy, with and without USP. After [33,180].

Fig. 7a shows the micrographs of solidified Al-Cu-Zr-Ti alloy samples, demonstrating the grain refinement effect before and after USP in bulk melts under resonant vessel dimensions (*L = λ*). As expected, the grain structure without USP in Fig. 7a was relatively coarse and irregular in shape. The average grain size for the untreated sample was measured to be approx. 260 µm. On the other hand, samples treated with USP demonstrated significant grain refinement with more than 50 % reduction in grain size, down to almost 120 µm within 60 s of treatment. The grains were much finer and more uniform as a result of enhanced heterogeneous nucleation of new grains within the melt and also through fragmentation of existing grains [180].

Fig. 7b shows the actual impact of USP on the grain structure of an alloy billet produced by DC casting in an industrial pilot scale facility. In this case, the partitions were installed in a launder at *L = λ* to achieve resonance conditions and maximize the residence time. Without USP, the billet (having length of 1 m and diameter of 152 mm) exhibited feathery or columnar grain structures associated with poor grain refinement and undesirable mechanical properties. When USP was applied, feathery regions were suppressed, and finer equiaxed grains dominated the billet cross-section, indicating improved grain refinement. It should be noted that the residence time inside the cavitation zone was significantly increased by 70 %, as predicted by our advanced numerical models in the case of partitions compared to non-partition launder configuration [213]. It has also been confirmed that the inclusion of Zr in the alloy leads to the formation of Al_3_Zr particles, which, upon fragmentation by cavitation, act as a potent nucleant for Al grains [197]. These experiments suggest that the observed grain refinement may be due to the fragmentation of primary intermetallics and nucleation of Al on them, facilitated by their interaction with cavitation-induced SWs during USP [33].

## 4 Metals additive manufacturing: Ultrasonic atomization for feedstock production

Atomization is a process of disintegrating liquids into fine droplets. It is a widely adopted technology across industries where precision, consistency, and material performance are paramount. Its applications span pharmaceuticals, energy, food processing, and advanced manufacturing, each exploiting controlled droplet formation to achieve specific outcomes. For example, in drug delivery, inhalers rely on atomized aerosols for targeted lung deposition [214], combustion systems optimize fuel efficiency through finely atomized sprays [215], while the food industry produces powdered flavours and instant products with tailored solubility [216] while coatings and paints also depend on uniform particle distribution for flawless finishes [217,218]. Central to these applications is the ability to govern droplet size, morphology, and dispersion, a capability that atomization markedly provides [79,81,154,[219], [220], [221], [222], [223], [224], [225], [226], [227]].

Similarly, within additive manufacturing (AM), atomization plays a pivotal role in providing suitable powder feedstock for processes such as laser powder bed fusion (LPBF), directed energy deposition (DED), selective laser melting (SLM) and electron beam melting (EBM) as the quality of AM produced parts is intrinsically linked to the particle size distribution (PSD) of metallic powders [82,85,[228], [229], [230], [231], [232]]. For example, ideal feedstock in various aluminium alloys requires spherical particles with a narrow size distribution (e.g. 15 – 45 µm for LPBF) to ensure optimal flowability, packing density, and melting behaviour. Deviations in shape or size can lead to defects such as porosity, uneven sintering, or mechanical weaknesses in final components.

Traditional methods like gas or water atomization often yield inconsistent powders, leading to defects in printed components. For example, gas atomization often leads to the incorporation of argon bubbles in the powder which are transferred to the component [233]. Ultrasonic atomization commercialized by Amazemet (Poland) offers a promising alternative, utilizing acoustic cavitation to generate fine, spherical metal powders that are pore free. In order to optimize and fully exploit this technology for tailored droplet/particle characteristics and enhanced performance, understanding the fundamental atomization mechanism becomes crucial. In this section, we will discuss a series of experiments conducted that elucidate the process mechanism observed during atomization of liquid droplets and production of Al feedstock particles. Prior studies hypothesized three competing mechanisms driving ultrasonic atomization; cavitation (bubble implosions breaking the liquid interface) [81], capillary waves (surface instabilities from acoustic vibrations) [234], and their conjunction (synergistic interplay) [78] but the debate persisted due to different mechanisms dominating under different ultrasonic intensities, liquid flow rate and atomizer system, or the absence of detectable cavitation [235]. Direct visualisation of the governing mechanism during atomization of aluminium alloys was further limited by the inability to visualize cavitation dynamics in opaque molten metals, unknown correlation between cavitation and capillary wave instability, and a lack of empirical data linking process parameters (e.g. amplitude) to particle characteristics. To address these gaps, we developed a multi-modal experimental framework by conducting high-speed optical imaging, synchrotron X-ray visualization, and acoustic emission measurements of a liquid droplet ultrasonically excited on a sonotrode tip. Water served as a transparent analogue for molten aluminium (refer to Section 3), enabling *in-situ* observation of cavitation bubbles and capillary wave dynamics. For molten aluminium, an Amazemet ultrasonic atomization system with a carbon fiber plate sonotrode attached to a 60 kHz transducer was employed, under inert conditions to mitigate oxidation [236].

**Fig. 8:**
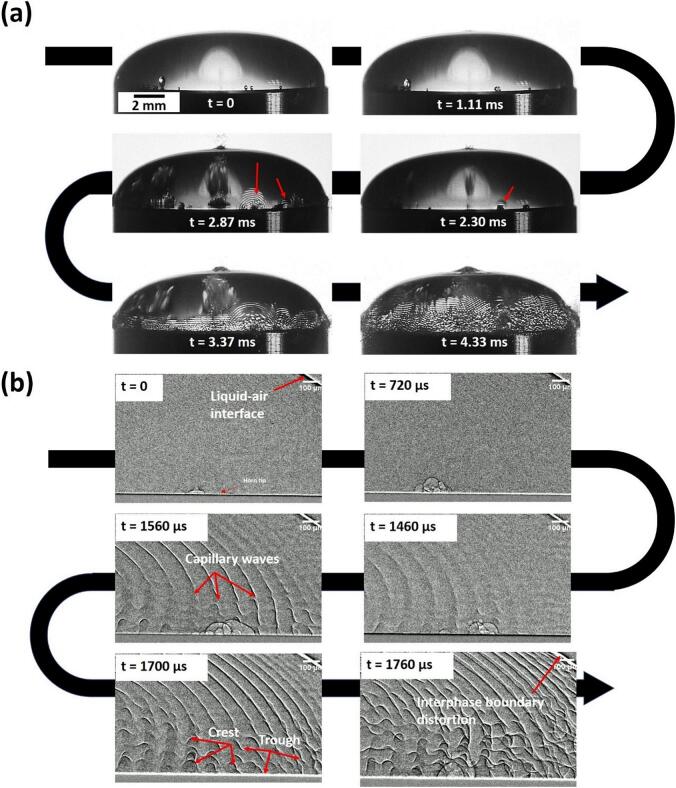

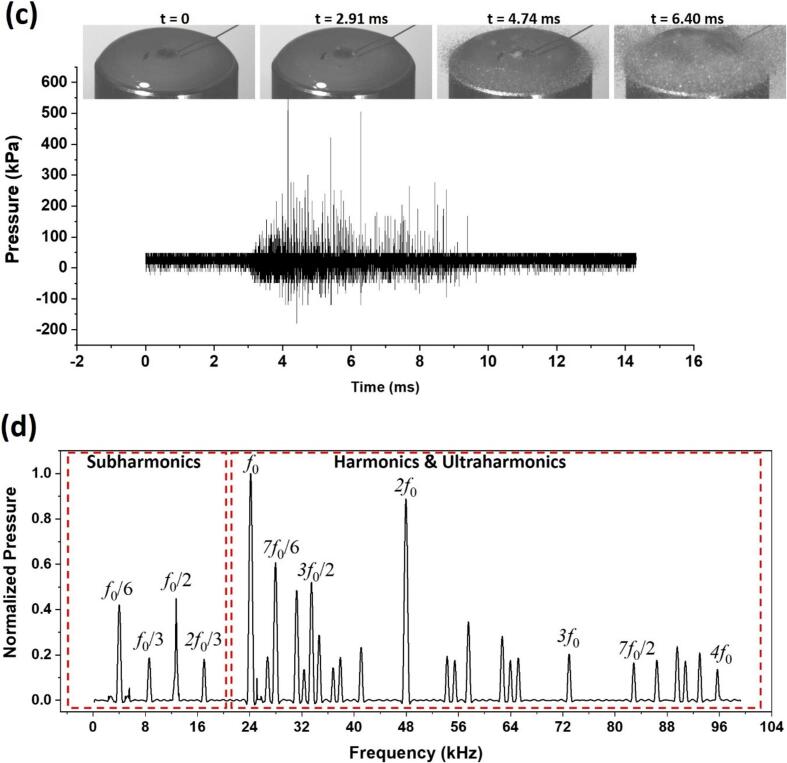
In-situ (a) optical and (b) X-ray high-speed imaging showing the evolution of atomization of water droplet on an ultrasonic horn, and acoustic pressure emissions captured during droplet formation in (c) time and (d) normalized pressure-frequency spectrum. After [236,240].

The temporal sequence shown in Fig. 8a from *t* = 1.11 ms to *t* = 4.33 ms captured the evolution of a water droplet under ultrasonic excitation. Initially, axisymmetric capillary (planar) waves (λ ≈ 105 µm, corresponding to the 24 kHz incident frequency from the acoustic source) form, but as they interact with spherical waves (λ ≈ 208 µm, corresponding to the subharmonic frequencies of 12 kHz) as imprints of SWs (marked by arrows) generated by repetitive bubble collapse in the vicinity of the liquid dome, they transition into chaotic patterns distorting the liquid–air interface. By 4.33 ms, this interference triggered ligament formation and droplet ejection, corroborating the conjunction theory where SWs amplify capillary instabilities. We also observed the atomization process using *in-situ* X-ray synchrotron visualization, complementing the high-speed optical imaging studies (Fig. 8b). This helped us to resolve the ambiguities in how cavitation directly influences wave characteristics and droplet formation. The X-ray data captured the nucleation and coalescence of microbubbles (45 – 200 µm) on the sonotrode surface, followed by the propagation of capillary waves (1.2–1.8 m/s) that became distorted near cavitation sites. These distortions culminated in droplet pinch-off (with size approx. 42 µm), with ligaments stretching and tearing off, consistent with cavitation-induced liquid jetting [237]. Notably, the droplet size correlated with half the capillary wavelength, while transition to subharmonic frequencies (associated with the periodicity of SWs) from the fundamental during bubble collapse confirmed that cavitation-generated SWs destabilized interphase boundary. Building on these qualitative observations, we quantitatively deciphered the role of cavitation and SWs by synchronizing high-speed imaging with acoustic measurements.

Time domain analysis (Fig. 8c) revealed transient pressure spikes (>500 kPa) between *t* = 1.94 and 4.74 ms, temporally aligning with cavitation collapses (source of high-energy SWs) and droplet ejection (3–5 m/s). The pressure–time plot further showed that the periodicity of these transient pressure spikes (associated with subharmonic frequencies) resulted from major peaks (indicative of SWs resulting from bubble collapse) in the acoustic emissions. Minor peaks, instead, were mainly associated with the fundamental frequency. The frequency domain spectra (Fig. 8d) showed subharmonic (2*f*_0_/3, *f_0_*/2, *f_0_*/3 and *f*_0_/6), harmonic (2*f*_0_, 3*f*_0_, 4*f*_0_), and ultraharmonic (3*f_0_*/2, 7*f*_0_/2, 7*f*_0_/6) peaks. The *f*_0_/2 subharmonic directly correlated with capillary wavelength doubling, driven by SWs from periodic bubble implosions. The subharmonic peaks in the spectrum are a direct measure of the energy released during implosive events [[182], [183], [184],^238^], and are linked to the transition from stable axisymmetric planar waves to unstable capillary waves, formed on the interphase boundary upon superimposition with the omnidirectional cavitation-induced SWs. The appearance of subharmonics at lower fractions, such as 2*f*_0_/3, *f*_0_/3, and *f*_0_/6 further suggests that the liquid exhibits resonant behaviour at specific modes revealing the characteristic of Faraday waves [239]. These frequencies also indicate that the transition from harmonic to subharmonic frequencies follows a systematic pattern driven by cavitation activity. Ultraharmonics, on the other hand, reflect higher-order microbubble cluster oscillations that can also result in harmonics from the subharmonic frequencies, generating periodic shock fronts that cause capillary wave formation. The interaction of SWs with the liquid–air interface is believed to introduce nonlinearities into the system that alter the characteristics of capillary waves, leading to droplet destabilisation and breakup.

**Fig. 9:**
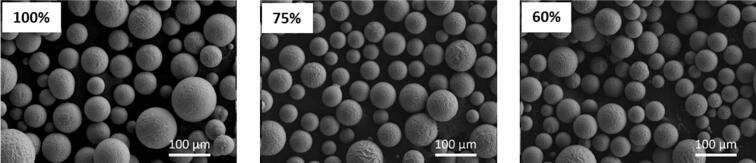
SEM micrographs of particles produced by atomization of pure liquid Al at different vibration amplitudes of ultrasound. After [236].

The atomization of liquid Al revealed that the vibration amplitude of the sonotrode critically dictates PSD and their morphology. As shown in Table 3, reducing the null-to-peak amplitude from 9.9 μm (100 % input power) to 8.55 μm (60 %) narrows the volumetric span (D_9_*_0_*−D_1_*_0_*) from 61.5 μm to 40.9 μm, with the median particle size (D_5_*_0_*) decreasing from 41.7 μm to 31.8 μm. This reflects enhanced uniformity, as lower amplitudes moderate cavitation intensity, producing smaller, more homogeneous droplets/particles. Notably, the count-based PSD reveals that over 50 % of particles (D_5_*_0_*) remain consistently fine (22–24 μm across all amplitudes), emphasizing a positively skewed distribution favouring finer particles, which improved packing density (a key advantage for AM). SWs generated during controlled bubble collapses likely induce strong perturbations in the molten film, destabilizing capillary waves and amplifying local instabilities. This disruption facilitates ligament thinning and subsequent pinch-off, leading to the formation of near-spherical particles as previously observed in the case of glycerol experiments, where SWs interactions influenced ligament ejections and droplet formation dynamics [236,240]. However, the *in-situ* visualization of liquid Al atomization remains to be examined. At higher amplitudes, aggressive cavitation may paradoxically coarsen particles due to chaotic bubble interactions or droplet coalescence, as evidenced by the wider volumetric spread at 100 % power. SEM analysis (Fig. 9) confirmed defect-free, satellite-less particles, attributable to SWs-driven rapid solidification that minimizes gas entrapment. By tuning amplitude to balance cavitation energy and SWs dynamics, the process achieves tailored feedstock with optimized flowability and packing density, essential for precision AM techniques such as DED and LPBF.

**Table 3:** Particle size by volume and number distribution of aluminium powders produced using ultrasonic atomization [236].

**Input power /Amplitude**	**Volumetric (µm)**			**Count (µm)**		
	Dv_10_	Dv_50_	Dv_90_	D_10_	D_50_	D_90_
100 % / 9.9 μm	21.3	41.7	82.8	15.2	24.5	42.7
75 % / 9 μm	18.0	32.4	64.4	12.4	22.1	34.9
60 % / 8.55 μm	18.2	31.8	59.1	12.8	22.4	34.6

## 5 Production of 2D nanomaterials: Cavitation induced exfoliation of graphite

The remarkable properties of 2D nanomaterials, especially graphene, have sparked intense research into innovative synthesis techniques that overcome the limitations of conventional methods. Graphene, discovered in 2004 [241], has since been named as a wonder material due to its extraordinary properties with excellent electrical and thermal conductivity, high mechanical strength and chemical activity, while being flexible and transparent [242]. These properties position graphene as a material with vast potential in fields ranging from electronics, energy storage, sensors to biotechnology, water purification and composite materials [[243], [244], [245]]. However, realizing the full potential of graphene relies on developing efficient, scalable, and sustainable synthesis methods to produce high-quality material with minimal defects [[246], [247], [248], [249]]. Several techniques have been utilized over the years for producing graphene ranging from mechanical exfoliation, chemical exfoliation, chemical reduction of graphene oxide and chemical vapour deposition (CVD) [61,250]. But, these conventional synthesis methods face significant limitations in terms of dispersing solvents used, which are often toxic, environmentally harmful, and expensive such as dimethyl sulfoxide (DMSO), N,N-dimethylformamide (DMF), N-methyl-2-pyrrolidone (NMP), tetramethylurea (TMU), tetrahydrofuran (THF), raising concerns about safety, contamination, and environmental impact [251,252]. Additionally, scalability and production costs remain significant challenges for methods like CVD and mechanical exfoliation, constraining industrial-scale implementation [253]. Thus, these limitations emphasize the need for alternative synthesis methods that balance quality, scalability, and environmental impact.

Ultrasonic assisted liquid phase exfoliation (ULPE) offers a promising method for the large-scale production of 2D materials with large surface areas, addressing many of the limitations associated with conventional synthesis methods [[254], [255], [256]]. This method was first tested by Coleman et al. in 2008 [62,257], and since then has gained success with additional complementing techniques such as high-shear mixing [258,259]. In most cases, these complementary techniques are used for dispersion of chemically exfoliated graphene, although ULPE has been demonstrated to be a powerful means for exfoliation by itself [68]. ULPE utilizes energetic cavitation bubbles to facilitate material exfoliation through powerful shear forces generated by both inertial and non-inertial cavitation phenomena. Inertial cavitation produces high energy SWs and liquid-jets from bubble implosions, while non-inertial generates rapid oscillating forces that contribute to the exfoliation process via micro-streaming and alternating compressive and tensile pressures. Nonetheless, despite the widespread attempts at ultrasonication for graphene production, the fundamental mechanisms have remained incompletely understood, which hinders the uptake of this technology. In this section, we review a series of experiments from our recent work on understanding the exfoliation mechanism of graphite through *in-situ* visualization and acoustic pressure measurements, followed by observations into the production of stable graphene dispersions using green solvents under single and dual frequency setups.

*In-situ* observations of exfoliation were conducted under both a single bubble (controlled cavitation) and cloud of bubbles (continuous cavitation) as described in Section 3, using a Shimadzu HPV X2 high-speed camera at 400 kfps with laser illumination (resolution of 400 × 250 pixels). Acoustic emissions were recorded synchronously with high-speed visualization using a calibrated FOH positioned below the sonotrode tip. Production of graphene flakes involved ultrasonicating graphite powder in DIW and eco-friendly solvents such as water/ethanol mixtures (DIW:EtOH) and water/isopropyl alcohol mixtures (DIW:IPA). Ultrasonic frequencies, including both low frequency (Lf) of 24 kHz and high frequency (Hf) of 1.174 MHz frequencies were employed to enhance the exfoliation process under optimized conditions of sonication power, vessel dimensions, sonotrode size, duration and temperature. Finally, to characterise the produced graphene flakes, Ultraviolet–visible (UV–Vis) spectroscopy and Raman spectroscopy were used to assess the quality, thickness, and yield of the graphene. Scanning electron microscopy (SEM) and high-resolution transmission electron microscopy (HR-TEM) were employed for morphological investigations, i.e. to determine the size, thickness, and number of layers of the exfoliated graphene flakes. The yield of produced graphene was estimated as the ratio of the final concentration of graphene obtained after ULPE followed by centrifugation to initial graphite concentration [68,71,260].

**Fig. 10:**
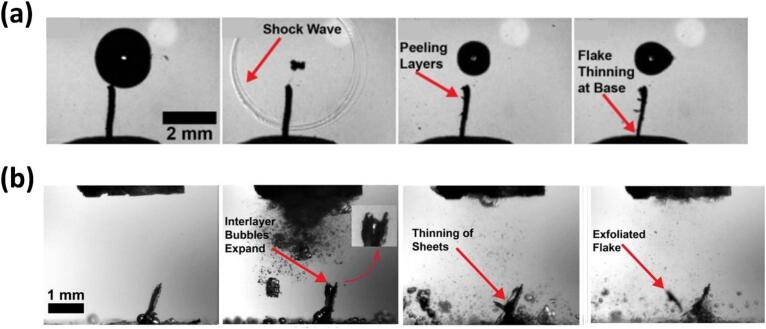

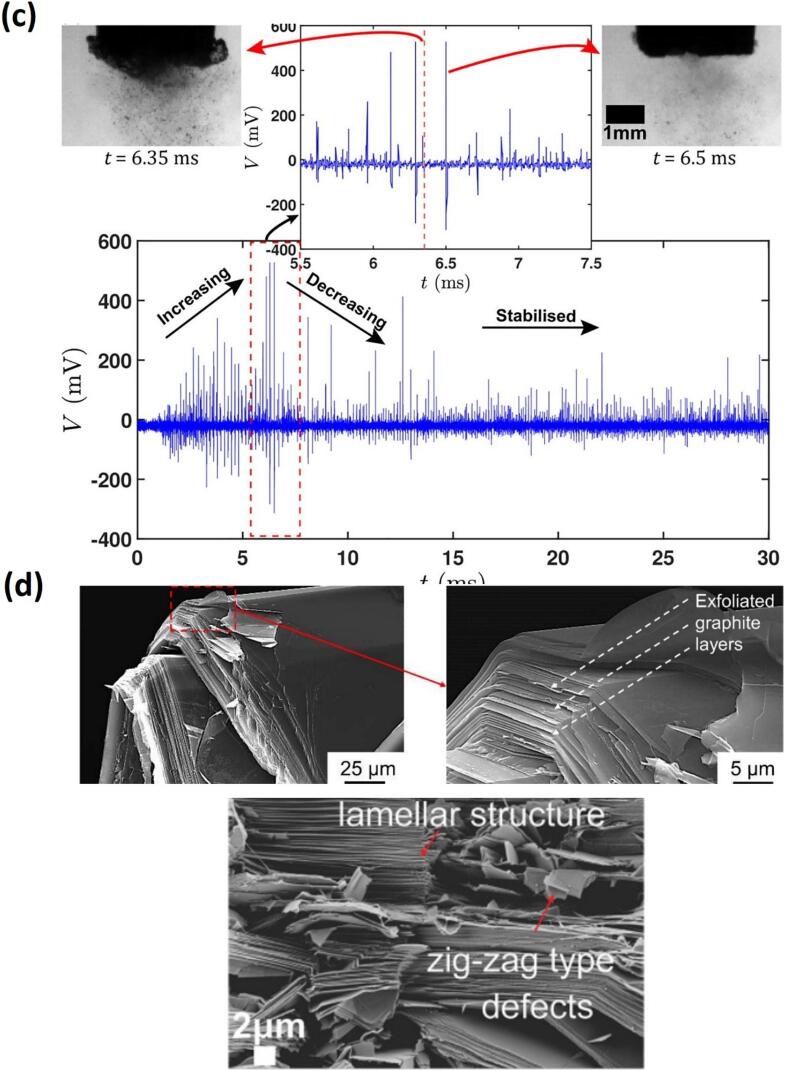

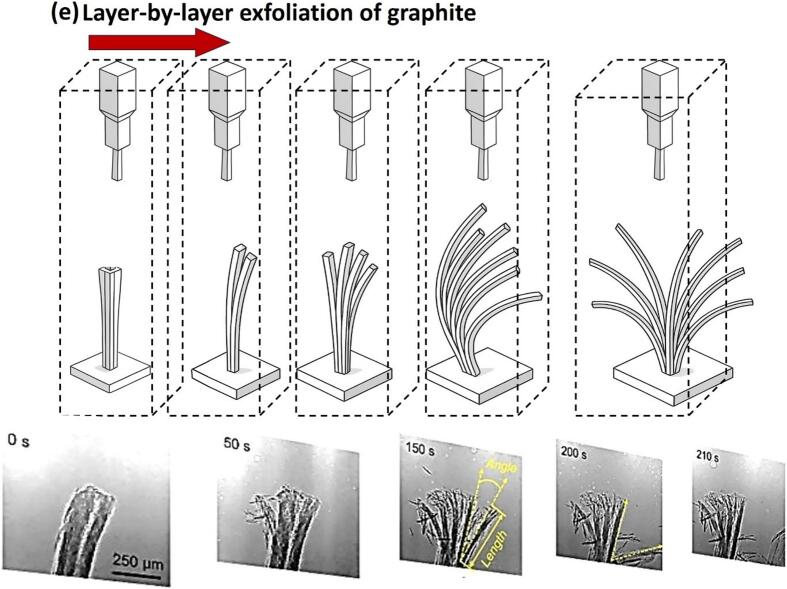
In-situ images showing graphite exfoliation by (a) single and (b) cloud of bubbles, (c) synchronized capturing of acoustic SWs intensity causing graphite proliferation and SEM images showing graphite structure (d) after ultrasonic treatment (top) and defects within exfoliated flakes (bottom), and (e) schematic (top) and X-ray image sequence (bottom) showing the layer exfoliation dynamics of a thin graphite. After [64,66,261].

Fig. 10a shows the image sequence of the contactless interaction of the SWs emitted from a single bubble collapse, widening and peeling the bulk graphite layers [261] in a motion that resembles petals blooming, the so-called “flowering” manifestation (schematically shown in Fig. 10e) [66]. The SWs not only widens the tip but also gradually thins the lower section, decreasing the graphite thickness with multiple SWs interactions. Similar features were also observed in case of bubble cloud collapses for proliferation of layer tearing off the graphite flakes within the cavitation zone (i.e. under a sonotrode tip) as shown in Fig. 10b. The experiment ensured the cavitation zone did not directly interact with the graphite flake by increasing the distance to ∼ 2.5 mm. It was found that the liquid-jet formed with speeds of up to 80 m/s from the implosion of a larger bubble, coalesced with non-inertial cavitation at the base separating the graphite layers resembling the flipping of the pages in a book. SWs with a pressure magnitude up to 5 MPa were revealed to initiate and propagate layer delamination. Additionally, non-inertial cavitation bubbles in the range 65 – 80 μm were shown to vigorously oscillate between the split layers, generating alternating pressures in the range of −35/+85 kPa expediting exfoliation in a fatigue manner via the so-called “branching” manifestation [66]. This process led to multilayer separation and thinning of the graphite sheets, influenced by inertial cavitation and acoustic streaming forces. Fig. 10c supported the camera observations where layer tearing proliferated during the initial sonication period, with cavitation intensity increasing until ∼ 7 ms followed by a drop in acoustic signal. The synchronized setup confirmed that the collapse of a fully developed bubble cloud at 6.5 ms coincided with a large signal peak, likely releasing multiple SWs that expedited graphite layer exfoliation. The half of time domain plot showed stabilized acoustic emissions with lower signals, reflecting the establishment of a smaller cavitation zone. Fig. 10d shows the SEM images of a graphite flake subjected to ultrasonic treatment. Prior to treatment, the flake exhibited a rough, uneven surface texture with multiple stacked layers surface. After the treatment, the graphite flake underwent significant exfoliation resulting in a fragmented and delaminated appearance (Fig. 10d − top). A close look at the exfoliated flake showed distorted topological defects alongside split graphite layer bundles with a thickness of 10–20 μm, likely produced by continuous and cyclic SWs bombardment (Fig. 10d − below).

Building upon fundamentals, and the realization of the importance of SWs on the exfoliation process, we first conducted experiments with Lf ULPE (where SWs emissions were expected to be prominent) to gauge the degree of exfoliation as a function of temperature involving both characterization studies and acoustic pressure measurements [65]. It was concluded that ULPE process at 40 °C at the studied input powers of 50 and 60 % (does not affect the quality of exfoliation but rather offers flexibility to the process) in pure DIW for 2 h reduces the thickness of graphite layers to high-quality few-layer graphene (FLG) exhibiting an area close 0.6 μm^2^ with some induced edge defects which are unavoidable in ULPE processes. It was further deduced that the width of the SWs emission peak can serve as a valuable tool for *in-situ* monitoring and refining of the ULPE process [64]. While opting for lower temperature settings might seem advantageous for generating a strong shock pressure field to enhance ULPE, a trade-off is required. This involves weighing the quantity of emitted SWs against the size of the cavitation zone (or bubbly cloud) formation, ensuring an optimal interaction that maximizes exfoliation efficiency, as also previously explained in Fig. 4c.

**Fig. 11:**
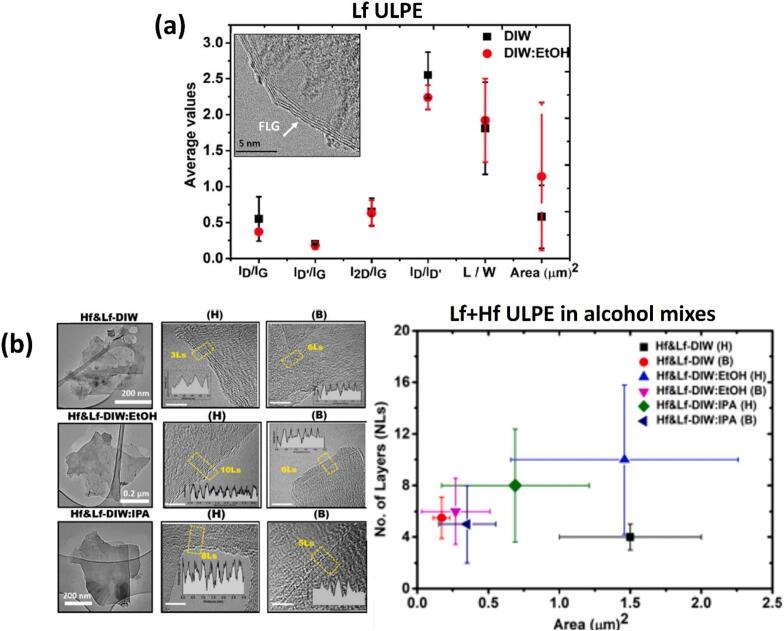

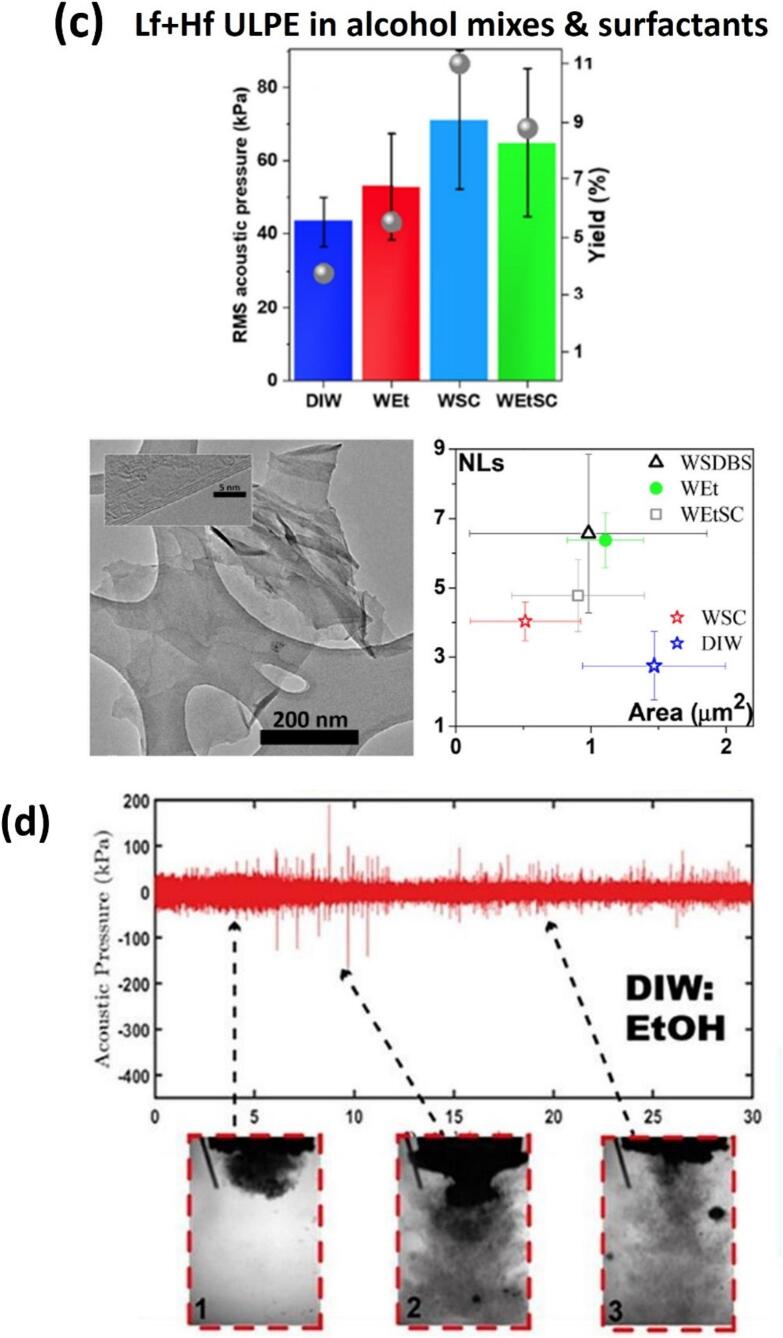
(a) The average values of ID/IG, ID’/IG, I2D/IG, ID/ID’, L/W and Area (with error margins) identified from Raman and TEM studies for the interrogated graphene flakes observed in both DIW and DIW:EtOH. (b) Typical TEM images of graphene flakes (low and high resolution) and statistical information on the number and area of exfoliated layers for both the H and B sonotrodes in DIW, DIW:EtOH, and DIW:IPA. (c) RMS acoustic pressures estimated and plotted with error bars (top) along with the measured yield (grey spheres, right axis) with TEM image at low and high resolutions (HR, inset) of a typical FLG flake obtained after ULPE process in the WSC solution (bottom), and (d) Synchronized imaging and acoustic pressure obtained for DIW:EtOH, showing cavitation dynamics at different time instant. After [64,67,262].

Fig. 11a features both the qualitative and quantitative analysis of exfoliated FLG in DIW and DIW:EtOH using the Lf setup, based on the parameters I_D_/I_G_, I_D’_/I_G_, I_D_/I_D’_ (quality), I_2D_/I_G_ (thinning effect of graphite), L/W and Area (μm)^2^ (size) identified from Raman and TEM measurements. Raman remains the most widely accepted and reliable tool for assessing the structural quality of graphene flakes, especially for evaluating defect density and flake morphology. The I_D_/I_G_ ratio serves as a direct indicator of the degree of disorder, with a lower ratio typically reflecting fewer structural defects. On the other hand, I_D_/I_D′_ ratio indicate the presence of edge-type rather than basal-plane defects. The prevalence of edge-type features is also consistent with the reduction in lateral flake size during sonication, which increases the relative contribution of edges to the Raman signal. The best results were obtained with a mixture of DIW and EtOH to produce higher quality (3–5) FLG with a yield twice that of DIW, with average flake area close to ∼ 1.15 μm^2^ and stability of ∼ 78 % over a duration of six months [67]. We observed that DIW as a liquid medium induced defects due to the greater impact of SWs interactions, whereas the mixture of DIW and EtOH was, in-part, proposed to balance generating less aggressive SWs (due to physical properties of alcohol-water mixture in equal volumes) impacts to exfoliate layered materials, as well as ensuring minimal surface damage, which promotes the production of pristine graphene and would increase applicatory uses of the exfoliated nano-sheets. Lf acoustic emissions resulted in the formation of larger bubbles or bubbly clouds, measuring a few hundred microns in size, with short lifecycles and powerful SWs upon their collapse, effectively loosening the tightly stacked graphite flakes. On the other hand, the Hf source generated smaller bubbles (few microns in size) that vigorously oscillated, rather than imploding, in a more stable (extended life cycles) manner, which infiltrated within the loose flakes, offering a “gentler exfoliation” of graphite by working between preliminary split and the expanded layers. Consequently, the synergy between these two cavitation regimes proved to be advantageous for both the quality and quantity of the exfoliated graphene [64].

Having identified the presence of SWs emissions by imploding cavitation bubbles and knowing that these SWs are instrumental in exfoliation of 2D materials, we implemented dual frequency ULPE utilizing different sonotrode sizes to produce graphene in environmentally friendly solvents. The findings in [67] indicated that under the sonotrode tip, cavitation development in DIW produces a more confined cavitation cloud, which is in contrast to DIW:IPA and DIW:EtOH mixtures, where both produced a much larger spatial distribution of cavitation bubbles, including additional, and larger sized satellite bubbles or bubbly clusters. Furthermore, both DIW:EtOH and DIW:IPA produced tiny “mist” cavitation bubbles which further aided in enhancing the cavitation zone, in addition to facilitating exfoliation of graphite (Fig. 11d). Further cavitation analysis revealed about 20 % larger measured *P*_RMS_ for dual frequency setups, particularly with the addition of EtOH or IPA, corresponding to greater graphene yields (approx. twice). It was shown that the larger size sonotrode (twice the diameter) can reduce the processing times by half while maintaining the same quality and yield levels. The similar pressures for both liquids indicated that the bell (B) shaped sonotrode of 40-mm in diameter produced comparable yields to the stepped horn (H) of 20-mm in size due to its double emitting surface that enlarges the cavitation zone and increases the amount of SWs. This is in combination with the role of the green solvents, which can further enhance the exfoliation efficiency of the cavitation zone. The different combinations of water, ethanol and green surfactants (such as cholic acid sodium salt (SC) and dodecylbenzene sulfonic acid sodium salt (SDBS)) were trialled to produce FLG flakes in our original dual frequency ULPE setup to investigate the effect of the medium onto the cavitation mechanism, which controls and affects the exfoliation of graphene [262]. It was discovered that different solutions promoted different cavitation patterns, which in turn influenced the final FLG flakes size, thickness, concentration and stability. The structural peculiarities of as-obtained FLG flakes were confirmed through characterization by Raman, UV–vis spectroscopies and HR-TEM. The characterization results (Fig. 11b) of the graphene samples, supported by the acoustic pressure analysis (Fig. 11c − top), showed that the use of DIW:IPA and DIW:EtOH represent efficient, eco-friendly solvents for producing high-quality FLG flakes with tailored thicknesses (4–10 Ls), area of flakes (0.3–1.5 μm^2^) (with DIW:EtOH producing significantly larger flakes than DIW:IPA), good yield (∼6%) and stable suspensions lasting over six-months (∼70 %), offering flexibility in a wide-range of applications such as using graphene as a biofriendly carrier for cancer treatment and electrodes for solar cells as demonstrated by our group [67,263,264]. The yield obtained in water and the other green solvents increased by almost 3 times following the addition of surfactants such as SC and SDBS correlating well with *P*_RMS_ data as seen in Fig. 11c (top). For example, WSC showed the highest yield (∼11 %) but also a higher defect level and smaller flakes, which is linked to higher intensity SWs that fragmented the graphite. In contrast, WEtSC solution, despite potentially having comparable *P*_RMS_ to other solvents, provided a better balance of yield (∼9%), lower defects, and larger flakes, along with significantly improved stability (retaining 78 % of flakes after 3 months). This suggests that while the overall acoustic power (represented by *P*_RMS_) might be similar in some cases, the specific cavitation pattern (e.g., aggressiveness of SWs, vigorously oscillating bubbles, extent of cavitation zone) differs based on the solution composition and critically influences the final graphene characteristics. Therefore, relying solely on *P*_RMS_ as a metric might be insufficient to fully differentiate the effectiveness of all conditions. In our studies, the yield of ∼ 10 % corresponds to a concentration of ∼ 0.04 mg/ml of FLG-enriched graphene supernatant, obtained after ULPE process from an initial graphite concentration of 0.4 mg/ml. It is important to note that the term ‘yield’ is often used qualitatively or statistically in the literature, referring to the proportion of FLG flakes observed within a scanned area during morphological investigations with the help of TEM and atomic force microscopy (AFM). Accordingly, yield is also sensitive to centrifugation speeds, in addition to the initial graphite concentration and exfoliation efficiency of the solvent. Lower centrifugation speeds tend to result in supernatants containing a higher proportion of heavier, bulkier graphitic materials and larger sized graphene flakes, which can inflate the measured weight of the filtered material at the expense of overall quality. Among all the liquid combinations, the mixture of water–ethanol and surfactant represented a very efficient green medium for dual frequency ULPE configuration with the high-yield (10 %) production of high-quality graphene (equal or less than 5Ls; reduced defects and at least 1 μm^2^ area) in less than 60 min with a very stable solution that retains 78 % of flakes in the suspension after 3 months as shown in Fig. 11c (top and bottom).

The importance of achieving stable graphene dispersions is pivotal for its commercialization, where end-users increasingly demand high-quality dispersions with high-throughput capabilities and extended shelf lives. Thus, ensuring that SWs produced during USP can effectively but gently exfoliate graphite into pristine graphene while minimizing damage to already separated flakes involves a multi-parameter optimization strategy. Firstly, combining Lf and Hf sources lead to a wider population and size distribution of cavitation bubbles. This enhanced cavitation activity allows for both effective initial delamination and subsequent gentle refinement of the graphene layers, significantly improving both the quality and yield of the exfoliated graphene. The dual frequency system also enlarges the cavitation cloud size and extends the boundaries of the cavitation zone, increasing the spatial distribution and lifecycle of satellite bubbles [64,67,265]. Secondly, the choice of solvent is critical as it directly influences cavitation behaviour and graphene quality. Unlike pure water, which induces defects due to more aggressive SW impacts, the addition of ethanol or isopropyl alcohol reduces the cavitation bubble impact and lead to less deleterious effects. These co-solvents facilitate the formation of a “cavitation mist” consisting of numerous tiny bubbles. This “mist” helps to cushion the aggressiveness of travelling SWs while promoting vigorous vibration of tiny bubbles that can infiltrate between loose interlayers of graphite, leading to gentle and uniform exfoliation [64,260,262,266]. Thirdly, precise control over processing temperature is essential for controlling cavitation activity and ensuring high-quality graphene. Higher temperatures lead to SW absorption by numerous bubbly clouds, reducing their intensity and making exfoliation inefficient, potentially causing “scissoring defects”. While lower temperatures restrict the cavitation zone, hindering efficient exfoliation despite high SW intensity. Therefore, an ideal temperature is somewhere intermediate involving a trade-off between achieving a large cavitation zone and generating sufficient SW emissions [65,189]. Lastly, real-time monitoring and post-exfoliation characterization are crucial in ensuring the gentle exfoliation while preserving the basal structure of graphene. Calibrated hydrophones measure acoustic pressures, allowing for the *in-situ* monitoring of cavitation activity and SW generation. For example, the width of the SW emission peak serves as an indicator for the uniformity of flake thickness and the completeness of the exfoliation process, offering a novel way to control and monitor in real-time [64]. Similarly, ultra high-speed imaging allows for direct observation of bubble dynamics and the various sono-exfoliation manifestations (e.g., “flowering,” “slicing,” “splitting,” “branching,” “page-flipping”) in real-time [66]. This visual feedback is invaluable for understanding the precise mechanisms at play and fine-tuning parameters to prevent damage. Post-exfoliation, characterization such as Raman spectroscopy qualitatively assesses structural defects and layers of graphene, TEM aids in evaluating morphology, layer count, lateral size and provides information related to the thickness, while UV–Vis estimates the concentration (yield) of exfoliated graphene and long-term stability of the dispersion [67,262,266].

## 6 Composite production: Ultrasound-assisted fiber impregnation

Fiber-impregnated composite materials have garnered significant attention across diverse industries, from aerospace to automotive, owing to their exceptional strength-to-weight ratio and design flexibility [[267], [268], [269]]. These materials are crucial for lightweighting and enhancing structural performance in demanding applications. However, realizing the full potential of these composites hinges on achieving uniform fiber distribution and optimal fiber–matrix interfacial bonding [270,271]. Recent studies have consistently highlighted that homogeneity in fiber dispersion and tailored fiber size are paramount for mitigating defect formation and maximizing the interfacial area, which directly translates to enhanced mechanical properties and overall material performance [[272], [273], [274]]. Traditional composite manufacturing methodologies, such as Resin Transfer Moulding (RTM) and Vacuum Assisted Resin Transfer Moulding (VARTM), while widely adopted, often encounter limitations in achieving critical microstructural characteristics [[275], [276], [277]]. These methods can also struggle with uniform fiber distribution and complete resin impregnation, particularly when dealing with complex geometries or high fiber volume fractions in highly viscous liquids and at elevated temperatures. This can lead to the formation of defects like voids, dry spots, and fiber misalignment, ultimately compromising the mechanical integrity and long-term durability of the composite [278,279]. Specifically, achieving complete resin wet-out of fibers is essential for stress transfer and preventing premature failure. However, the inherent high viscosity and surface tension of many polymeric resins, especially advanced thermoplastic melts, impede effective impregnation, resulting in porosity and reduced mechanical performance [280,281].

Ultrasound offers a compelling alternative as it can induce a range of physical phenomena that directly address the limitations of traditional methods [[282], [283], [284]]. This technology can significantly enhance fiber dispersion and resin impregnation through several key mechanisms [[285], [286], [287]]: (i) by effectively reducing the apparent viscosity of the resin and improving its flow characteristics, thus enabling better penetration into fiber network and (ii) in addition to viscosity reduction, ultrasound induced cavitation effects can increase the surface roughness and activity of the fibers, thereby enhancing their wettability and promoting stronger fiber–matrix adhesion. This improved wettability is critical for ensuring strong contact and robust bonding between the fibers and the resin. (iii) Furthermore, the induced acoustic streaming, SWs, and microjets generated by ultrasound can create localized fluid motion, promoting resin movement, separating clustered fibers, and facilitating the uniform dispersion of fillers and fibers throughout the matrix.

**Fig. 12:**
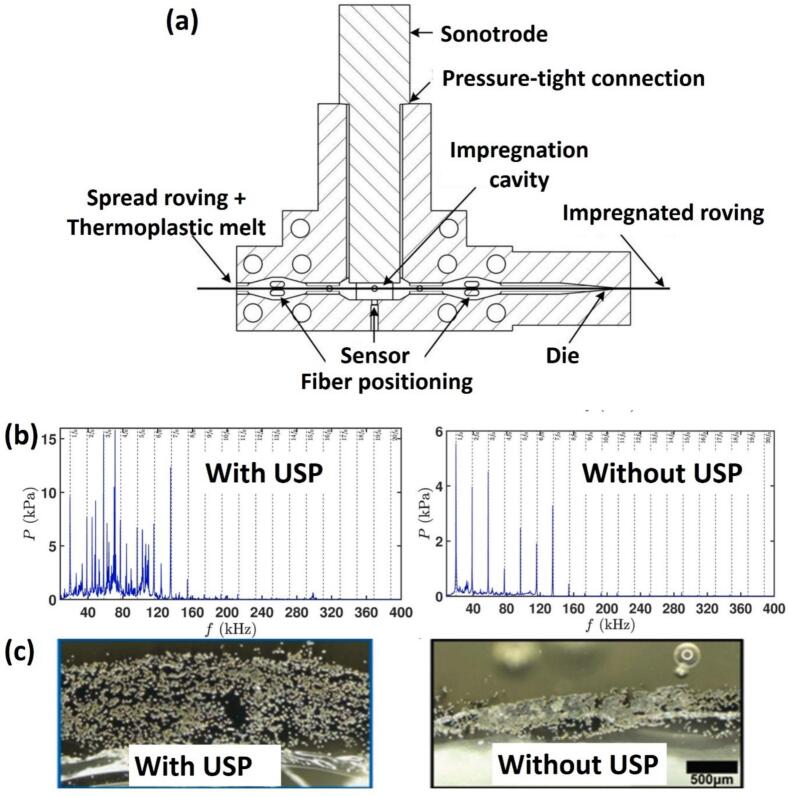

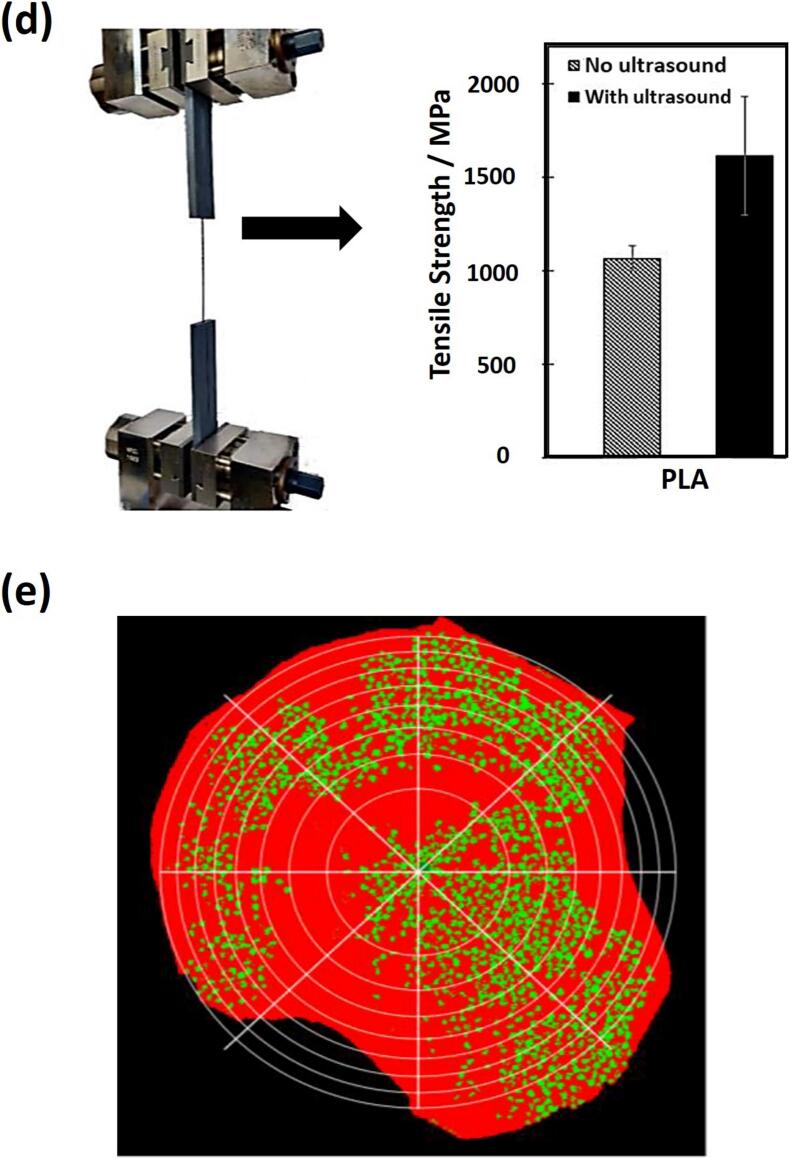
(a) Schematic representation of ultrasound impregnation cell, (b) acoustic spectrum captured with cavitometer submerged in PLA melt with and without cavitation, (c) cross-section images of impregnated roving with and without introduction of ultrasound into the melt batch, (d) tensile testing of PLA-fiber composite and (e) analysis of fiber impregnation through radial ring segmentation. After [288,289].

We conducted a series of experiments by inducing ultrasound into thermoplastic polylactide (PLA), a highly viscous polymeric melt, which allowed for a detailed characterization of the cavitation zone confined to the 2 mm depth under the tip of the sonotrode and evaluation of the effects of process parameters on the impregnation process. A melt bath setup designed to produced continuous fibre-reinforced 3D-printing filament from 24 K roving was employed along with a 20 mm diameter sonotrode operating at 19.5 kHz to introduce ultrasound into PLA melt as shown in Fig. 12a. The melt temperature was precisely controlled at 180 °C with the sonotrode positioned 5 mm from the bottom of the glass beaker. A high-temperature calibrated cavitometer (as previously described in Section 3 and in [174]) was also employed to measure acoustic emissions, positioned at a 45° angle and varied between 2 mm, 3 mm, and 4 mm distances from the sonotrode tip. Data was captured using a digital oscilloscope and processed using Fast Fourier Transform to obtain frequency domain data, with the background noise subtracted as described in [180]. The sonotrode amplitude was varied at 50 % and 100 % while acoustic pressure was measured, and the results were compared to similar measurements in water.

Fig. 12b represents the acoustic cavitation spectrum obtained for PLA at 50 % input power of ultrasound at a distance of 2 mm and 4 mm away from the sonotrode tip. The spectrum labelled ‘without USP’ was acquired 4 mm away from the tip and shows a non-cavitating regime, evidenced by the absence of broadband noise (no rise in the noise floor) and lack of subharmonic and ultraharmonic peaks. Although ultrasound was active, the local pressure field was insufficient to sustain cavitation. In contrast, the ‘with USP’ spectrum, recorded at 2 mm distance, exhibited characteristic signatures of developed cavitation such as harmonics, ultra-harmonics, and subharmonics along with the rise in broadband noise (as described in 2, 3, 4) indicative of bubble oscillations and SWs emissions emitted from non-spherical collapses (transient cavitation). It has been shown that for distances within the close proximity of the sonication tip (< 3 mm), the generated spectra exhibit prominent subharmonic peaks with a rise of the broadband floor indicating the strong presence of SW emissions from collapsing bubbles or bubbly clusters [184] in contrast to the non-cavitating melt where only the fundamental peak and corresponding harmonics were observed [288]. After the treatment, the roving was extracted and cut at the treatment position to assess the ultrasound effect through optical image analysis of sample cross-section (Fig. 12c). Following 3 s of treatment, significant improvements in fiber impregnation and distribution were observed compared to untreated roving. The fibers, which tend to cluster in untreated roving, were well-dispersed in the sonicated melt, forming a thicker boundary. Subsequently, tensile testing (Fig. 12d) was used to identify and compare the influence of ultrasound treatment on fiber impregnation, while the distribution was evaluated through segmentation process. For each experimental condition with and without USP, a total of 10 tensile specimens were evaluated. The testing was carried out using standard protocols, with the strain captured via extensometer to ensure accuracy and consistency. An approximate 50 % increase in strength was observed for the composite treated with ultrasound compared to the untreated one. The tensile strength values for the ultrasound-treated specimens showed a wider spread, with variability around ± 80 MPa, whereas the untreated samples exhibited much tighter clustering with fluctuations of about ± 25 MPa. This improvement in strength is attributed to the enhanced fiber wetting and dispersion achieved through ultrasonic treatment. The induced cavitation and acoustic streaming facilitated better infiltration of the polymer melt into the fiber bundles, reducing voids and improving fiber–matrix adhesion. The evaluation of fiber distribution involved segmenting cross-section images into angular triangles and radial circular rings (Fig. 12e). The findings indicate that the introduction of acoustic cavitation improves the fiber distribution especially with respect to the centre of the filaments.

## 7 Conclusions

The use of sophisticated *in-situ* experimental diagnostic tools enables researchers and industry professionals to uncover both the fundamental mechanisms and the application potential of USP. Obtaining detailed qualitative and quantitative insights into the cavitation dynamics is essential for advancing and fine-tuning ultrasound-assisted processing techniques across a wide range of metallic and non-metallic materials. This review advances our understanding of the behaviour of SWs and dynamics in USP, paving the way for significant developments in material science and engineering applications. The following conclusions can be drawn from our experimental studies summarized in this review:

1. Optimal ultrasonic processing conditions can be achieved through careful consideration and trade-off between the choice of liquid medium, temperature and transducer power that minimizes the cavitation shielding effect and promotes SW propagation
2. Fragmentation-assisted grain refinement of Al alloys is primarily driven by the repetitive interaction of intermetallic crystals with high energy SWs inducing high load-low cycle fatigue leading to their fracture. The fragmented crystals then act as secondary sites for grain nucleation
3. The efficiency of USP in DC casting can be improved by choosing resonant size launders that intensifies cavitation activity and acoustic flow patterns while maximizing residence time of the melt
4. Atomization of liquids on an ultrasonic horn system is triggered by the cavitation-induced SWs from periodic bubble collapses that interferes with vibration-induced capillary waves resulting in the formation of chaotic patterns followed by droplet pinch-off and ejection.
5. Defect free spherical Al particles can be obtained by adjusting vibration amplitude of ultrasound under inert atmospheric conditions
6. ULPE is primarily driven by the energetic activity of cavitation bubbles, where the violent collapse of transient bubbles generates shock waves and liquid jets that overcome the interlayer van der Waals forces in graphite to initiate exfoliation, while the oscillations of non-inertial bubbles and acoustic streaming further contribute to delamination and dispersion of graphene flakes.
7. Using green solvents in a dual-frequency ULPE setup, high-quality few-layer graphene can be produced with a yield twice that of water alone while being stable for over six months. A larger sonotrode size halves the processing times while maintaining quality, utilizing enhanced cavitation and acoustic pressures for scalable, sustainable production
8. Effective impregnation of fiber bundles within the thermoplastic melt is confined to the vicinity of the ultrasonic source, where intense cavitation activity complemented by local pressure surges from emitted SWs occur.
9. USP enhances uniform fiber distribution close to the centre of filaments leading to 50 % increase in tensile strength of the composite material

Despite the significant progress made towards understanding the fundamental mechanisms that govern these applications, the path to widespread industrial adoption is still paved with specific challenges related to scalability, process control, and material quality and, therefore, needs to be addressed in future studies. For example, in metal casting, current deployment of USP is restricted to pilot-scale processing volumes due to acoustic attenuation, making real-time control of cavitation and melt uniformity difficult in large scale continuous casting. Similarly, metal powder production struggles with precise control over particle size distribution and maintaining consistent quality at high throughput. In nanomaterial synthesis, high energy consumption and low yield are major hurdles. Likewise, in composite production, achieving uniform impregnation in high-fiber volume fraction systems and precise control over ultrasound-resin interaction is problematic.

Extending beyond these applications, USP is being increasingly applied across several other emerging and high impact fields. For example, in environmental decontamination and water treatment, USP can be harnessed for enhanced degradation of persistent organic pollutants such as per- and polyfluoroalkyl substances (PFAS), disinfection, and sludge treatment, offering a green alternative to conventional methods. USP also produces size-specific nanoparticles/nanosheets with tailored properties for electrode fabrication to be used in fuel cells, hydrogen storage systems and advanced battery technologies. In food extraction and processing, USP could significantly enhance the extraction yield of bioactive compounds and improve emulsification. Whereas, in biomedical applications, USP allows size-specific production of nanocarriers which are crucial for biodistribution and maximizing their therapeutic potential in targeted drug delivery. These diverse and emerging applications of USP prove that the fundamental principles that govern such processes are highly transferable. Success in one domain can directly or indirectly inform and accelerate advancements in seemingly disparate fields, accentuating the interdisciplinary power of ultrasonic processing.

## CRediT authorship contribution statement

**Abhinav Priyadarshi:** Writing – review & editing, Writing – original draft, Validation, Software, Methodology, Investigation, Formal analysis, Data curation. **Amanpreet Kaur:** Writing – original draft, Methodology, Investigation, Formal analysis, Data curation. **Mohammad Khavari:** Writing – review & editing, Visualization, Software, Methodology, Investigation, Formal analysis, Data curation. **Justin A. Morton:** Writing – review & editing, Visualization, Software, Methodology, Investigation, Formal analysis, Data curation. **Anastasia V. Tyurnina:** Methodology, Investigation, Formal analysis, Data curation. **Morteza Ghorbani:** Investigation, Funding acquisition. **Paul Prentice:** Writing – review & editing, Resources, Methodology. **Jiawei Mi:** Supervision, Resources, Methodology, Investigation, Funding acquisition. **Koulis Pericleous:** Writing – review & editing, Project administration, Funding acquisition. **Peter D. Lee:** Writing – review & editing, Project administration, Funding acquisition. **Dmitry G. Eskin:** Writing – review & editing, Supervision, Resources, Project administration, Funding acquisition. **Iakovos Tzanakis:** Writing – review & editing, Supervision, Resources, Project administration, Funding acquisition, Conceptualization.

## Declaration of competing interest

The authors declare that they have no known competing financial interests or personal relationships that could have appeared to influence the work reported in this paper.
